# A Novel Biotinylated Homotryptamine Derivative for Quantum Dot Imaging of Serotonin Transporter in Live Cells

**DOI:** 10.3389/fncel.2021.667044

**Published:** 2021-11-18

**Authors:** Ian D. Tomlinson, Oleg Kovtun, Ruben Torres, Laurel G. Bellocchio, Travis Josephs, Sandra J. Rosenthal

**Affiliations:** ^1^Department of Chemistry, Vanderbilt University, Nashville, TN, United States; ^2^Vanderbilt Institute of Chemical Biology, Vanderbilt University, Nashville, TN, United States; ^3^Neuroscience Program, Vanderbilt University, Nashville, TN, United States; ^4^Department of Pharmacology, Vanderbilt University, Nashville, TN, United States; ^5^Department of Chemical and Biomolecular Engineering, Vanderbilt University, Nashville, TN, United States; ^6^Department of Physics and Astronomy, Vanderbilt University, Nashville, TN, United States; ^7^Vanderbilt Institute of Nanoscale Science and Engineering, Vanderbilt University, Nashville, TN, United States

**Keywords:** serotonin transporter, ligand, quantum dot, labeling, single particle tracking

## Abstract

The serotonin transporter (SERT) is the primary target for selective serotonin reuptake inhibitor (SSRI) antidepressants that are thought to exert their therapeutic effects by increasing the synaptic concentration of serotonin. Consequently, probes that can be utilized to study cellular trafficking of SERT are valuable research tools. We have developed a novel ligand (IDT785) that is composed of a SERT antagonist (a tetrahydro pyridyl indole derivative) conjugated to a biotinylated poly ethylene glycol (PEG) via a phenethyl linker. This compound was determined to be biologically active and inhibited SERT-mediated reuptake of IDT307 with the half-maximal inhibitory concentration of 7.2 ± 0.3 μM. We demonstrated that IDT785 enabled quantum dot (QD) labeling of membrane SERT in transfected HEK-293 cultures that could be blocked using the high affinity serotonin reuptake inhibitor paroxetine. Molecular docking studies suggested that IDT785 might be binding to the extracellular vestibule binding site rather than the orthosteric substrate binding site, which could be attributable to the hydrophilicity of the PEG chain and the increased loss of degrees of freedom that would be required to penetrate into the orthosteric binding site. Using IDT785, we were able to study the membrane localization and membrane dynamics of YFP-SERT heterologously expressed in HEK-293 cells and demonstrated that SERT expression was enriched in the membrane edge and in thin cellular protrusions.

## Introduction

5-hydroxytryptamine (5-HT, serotonin) is a monoamine neurotransmitter involved in the modulation of a myriad of functions in the brain and periphery, including mood, appetite, sleep, memory and learning. The magnitude and duration of serotonin signaling is primarily controlled by rapid clearance of extracellular serotonin via the high-affinity serotonin transporter (SERT; *SLC6A4*) (Blakely et al., 1991; Ramamoorthy et al., 1993; Torres et al., 2003). SERT belongs to a family of twelve transmembrane-spanning domain neurotransmitter::sodium symporters (NSS) that harness the electrochemical gradient of Na^+^ and Cl^–^ to translocate substrate through a series of structural rearrangements from outward-open to inward-open conformation (Coleman et al., 2016, 2019, 2020; Cheng and Bahar, 2019). Alterations in SERT activity and expression have been implicated in several neuropsychiatric disorders, including anxiety, autism spectrum disorder (ASD), obsessive-compulsive disorder (OCD), attention deficit/hyperactivity disorder (ADHD), and major depressive disorder (MDD) (Kristensen et al., 2011). Accordingly, SERT is an important pharmacological target for widely prescribed selective serotonin reuptake inhibitors (SSRIs), including paroxetine, citalopram, and fluoxetine (Coleman and Gouaux, 2018), which are used to treat MDD, OCD, and anxiety disorders. Moreover, single nucleotide polymorphisms (SNPs) in the SERT gene can affect the expression, membrane trafficking and activity of the transporter and are enriched in families with neuropsychiatric disorders (Hu et al., 2006; Hahn and Blakely, 2007). SERT activity is tightly regulated at the post-translational level by a complex interplay of kinases, phosphatases, and numerous other SERT interacting proteins (Ramamoorthy et al., 1998; Bermingham and Blakely, 2016; Cooper et al., 2019; Quinlan et al., 2020). Consequently, methodologies that can dissect SERT regulation at the single transporter level represent a promising experimental avenue toward understanding the molecular basis of SERT-associated neuropsychiatric disorders.

Single-molecule fluorescence microscopy is an umbrella term for non-invasive imaging modalities that have emerged in the investigation of transmembrane protein trafficking and regulation, as they can access nanometer spatial and sub-second temporal scales (Rosenthal et al., 2011; Kusumi et al., 2014). Typically, an antibody, a peptide or a derivative of a drug or neurotransmitter are conjugated to a fluorophore and utilized as a probe for optical detection of a membrane protein. The fluorophore is usually separated from the targeting functionality via a spacer arm; this is often required to maintain biological activity of the targeting portion of the probe and may also aid in water solubility. Several dye-conjugated antagonists of neuronal receptors and transporters have been reported in the literature (Madras et al., 1990; Bakthavachalam et al., 1991; Jacobson et al., 1995; Rayport and Sulzer, 1995; Cha et al., 2005; Eriksen et al., 2009; She et al., 2020). However, many commonly used fluorescent dyes have low quantum yields and suboptimal photostability, thereby limiting their utility in experiments that require prolonged monitoring of protein membrane dynamics at video rates (Resch-Genger et al., 2008). Biocompatible colloidal semiconductor quantum dots (QDs) are an attractive alternative to dye fluorophores (Rosenthal et al., 2011). These highly fluorescent semiconductor crystals range in diameter from 2 to 10 nm, have a quantum yield that is significantly larger than many commercial dyes and a narrow, size-tunable, Gaussian emission spectrum. Their large absorption cross section is a continuum above the 1st excitation feature, enabling the use of a single excitation source for multicolor experiments. Many studies using antibody- and peptide-conjugated quantum dots in biological labeling of live cells have been reported (Dahan et al., 2003; Bouzigues et al., 2007; Lee et al., 2017; Bailey et al., 2018). The cornerstone of our QD labeling strategy has been the development of agonist- and antagonist-based fluorescent probes. Unlike the antibody-based approach, such ligand probes do not require a suitable extracellular epitope or an engineered tag in the extracellular domain of the target protein for binding; also, their affinity and binding properties may be modified by the incorporation of simple structural changes into the ligand (Tomlinson et al., 2019). To date, we have successfully developed antagonist-based QD probes to label SERT and other membrane-bound neuronal proteins, including structurally related dopamine transporter (DAT) (Kovtun et al., 2011; Chang et al., 2012b; Kovtun et al., 2015; Rosenthal, 2019; Tomlinson et al., 2019).

**FIGURE 1:**
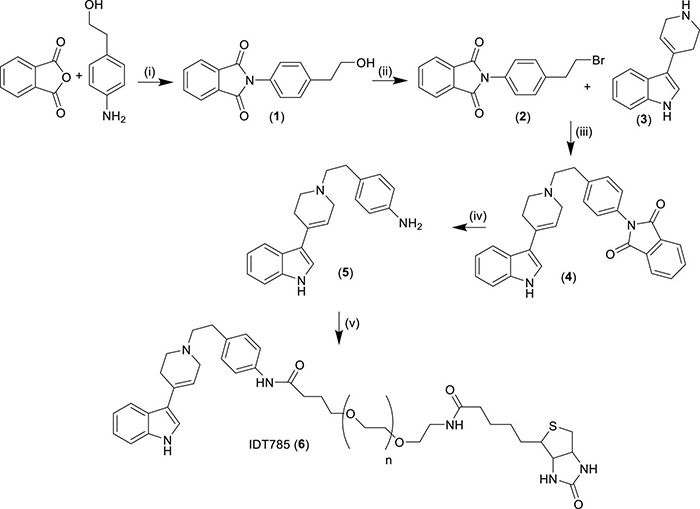
Synthesis of biotinylated SERT ligand IDT785. (i) Pyridine, reflux 18 h, yield = 48%; (ii) PPh_3_, triethylamine, methylene chloride, 18 h, yield = 57%; (iii) acetonitrile, triethylamine, reflux, 18 h, yield = 32%; (iv) (a) hydrazine hydrate, ethanol, 1 h, (b) methylene chloride, 18 h, yield = 70%; (v) biotin-PEG5000-SVA, methylene chloride, 18 h. Compounds in bold numbers: (1) = 2-(4-(2-hydroxyethyl)phenyl)isoindoline-1,3-dione, (2) = 2-(4-(2-bromoethyl)phenyl)isoindoline-1,3-dione, (3) = 3-(1,2,3,6-tetrahydropyridin-4-yl)-1*H*-indol, (4) = 2-(4-(2-(4-(1*H*-indol-3-yl)-3,6-dihydropyridin-1(2*H*)-yl)ethyl)phenyl)isoindoline-1,3-dione, (5) = 4-(2-(4-(1*H*-indol-3-yl)-3,6-dihydropyridin-1(2*H*)-yl)ethyl)aniline.

Over the past two decades, we have devised several strategies in probe development, including direct attachment of the ligand to a polymer on the QD surface via a covalent bond (Gussin et al., 2006) and ligands that incorporate a biotin moiety for non-covalent binding to streptavidin on the surface of the dot (Kovtun et al., 2011; Tomlinson et al., 2019). We have observed that conjugation of polyethylene glycol (PEG; PEGylation) to the QD surface may be required to overcome non-specific binding to the cell surface (Bentzen et al., 2005) and have incorporated PEG chains into our ligands for this purpose. Currently, we rely on commercially available streptavidin-conjugated QDs that display lower non-specific binding properties. The linker arm of choice in our probes has been a polyethylene glycol (PEG) chain attached to the antagonist via an alkyl spacer, with the biotinylated distal end of the PEG forming the point of attachment to the QD (Tomlinson et al., 2011). Recently, we have observed that the nature of the spacer between the PEG chain and the antagonist can have profound effects on non-specific coverslip binding (Tomlinson et al., 2019). Here, we report synthesis of a novel SERT ligand IDT785 (Figure 1) with an electron-rich phenylethyl spacer between the PEG chain and the antagonist. We study its ability to inhibit the human SERT transiently expressed in HEK-293T cells (hSERT), examine specific labeling of hSERT and SERT fused to yellow fluorescent protein (YFP) at the N-terminus with a combination of biotinylated IDT785 streptavidin-conjugated QDs (SavQD), and demonstrate that hSERT surface dynamics may be tracked with IDT785-QD conjugates.

## Materials and Methods

### Chemistry

^1^H NMR and ^13^C NMR spectra were obtained on a Brucker AV-400 (400 MHz) NMR spectrometer using deuterated chloroform as the solvent unless otherwise stated. Chemical shifts were expressed in parts per million and were measured relative to tetramethyl silane (TMS). Biotin-PEG-SVA consists of biotin attached to the terminus of poly dispersed PEG5000 whilst the other end of the PEG was terminated with a succinimidyl valerate (SVA) functionality that ultimately forms the point of attachment to the SERT antagonist. This reagent was obtained from Laysan Bio, Arab, AL. The mass of this reagent was assumed to be 5000 Daltons and this was used when calculating the quantities of reagents required to react with it. The progress of all the reactions was monitored by TLC, which was performed on aluminum sheets precoated with silica gel 60 (HF-254, Merck). The developed chromatograms were visualized using UV light (254–365 nm). Sorbtech silica gel (63–200 mesh) was used for preparative column chromatography. IDT307 was synthesized in house as previously described (Blakely et al., 2011). Reagents and solvents for the synthesis of IDT785 were ordered from Sigma Aldrich, Fischer Scientific and VWR, and used without further purification.

### Ligand Synthesis

The synthesis of IDT785 is outlined in Figure 1. Initially the spacer arm was synthesized by reacting 4-aminophenethyl alcohol with phthalic anhydride to yield the phthalimide (1) in a 48% yield. Then the alcohol was converted to a bromide utilizing an Appel reaction giving the bromide (2) in a 57% yield. The bromo intermediate (2) was coupled to the SERT antagonist (3) via a nucleophilic displacement of the bromide by refluxing in acetonitrile. This gave the phthalimide protected intermediate (4) in a 32% yield. Then the phthalimide protecting group was removed by treatment with hydrazine hydrate resulting in the amino intermediate (5) in a 70% yield. This was coupled to the NHS ester of the biotinylated PEG and the final ligand was purified by size exclusion chromatography on a Sephadex G10 column to yield the final ligand IDT785 (6) which was used without further purification.

#### 2-(4-(2-hydroxyethyl)phenyl)isoindoline-1,3-dione (1)

Phthalic anhydride (6 g, 0.0404 mols) and 4-aminophenethyl alcohol (5.54 g, 0.0404 mols) were mixed in pyridine (150 mL) and heated at reflux with stirring for 18 h (dos Santos et al., 2011). Then the solution was cooled and added to methylene chloride (200 mL). The solution was washed with copper sulfate solution (2 × 100 mL, 10%) then with deionized water (2 × 100 mL) and dried over magnesium sulfate. Then it was filtered and evaporated under reduced pressure. The product was semi purified by column chromatography on silica gel eluted with a gradient system running from ethyl acetate (50%) / hexanes (50%) to ethyl acetate (100%). The resultant semi pure product was then dissolved in methylene chloride (150 mL) and washed with hydrochloric acid (2M, 2 × 50 mL) and deionized water (2 × 100 mL) to remove unreacted 4-aminophenethyl alcohol. Then it was dried over magnesium sulfate filtered and evaporated to give pure product (1) as a white solid in a 48% yield. ^1^H NMR (CDCl_3_) δ 7.99–7.92 (m, 2H), 7.82–7.74 (m, 2H), 7.38 (s, 4H), 3.88 (q, 2H), 2.92 (t, 2H), and 1.66 (brs, 1H); ^13^C NMR (CDCl_3_) δ 167.34, 138.76, 134.38, 131.71, 129.92, 129.78, 126.66, 123.72, 63.43, and 38.83.

#### 2-(4-(2-bromoethyl)phenyl)isoindoline-1,3-dione (2)

2-(4-(2-hydroxyethyl)phenyl)isoindoline-1,3-dione (1) (5.2 g, 0.0195 mols) was dissolved in methylene chloride (100 mL) followed by triphenyl phosphine (5.64 g, 0.215 mols). The resultant solution was stirred at ambient temperature and N-bromosuccinimide (3.73 g, 0.0210 mols) was added portion wise, after which stirring was continued for 18 h at ambient temperature. Then the solvent was removed under reduced pressure and the product was purified by column chromatography on silica gel eluted with ethyl acetate (50%) / hexanes (50%) to give 3.7 g of (2) as a white solid in a 57% yield. ^1^H NMR (CDCl_3_) δ 7.98–7.92 (m, 2H), 7.82–7.78 (m, 2H), 7.42–7.34 (m, 4H), 3.59 (t, 2H), 3.22 (t, 2H); ^13^C NMR (CDCl_3_) δ 167.23, 138.80, 134.41, 131.68, 130.38, 129.39, 126.64, 123.74, 39.01, and 32.37.

#### 2-(4-(2-(4-(1*H*-indol-3-yl)-3,6-dihydropyridin-1(2*H*)-yl)ethyl)phenyl)isoindoline-1,3-dione (4)

3-(1,2,3,6-tetrahydropyridin-4-yl)-1*H*-indol (3) was synthesized as previously described (Tomlinson et al., 2005). 2-(4-(2-bromoethyl)phenyl)isoindoline-1,3-dione (2) (3.7 g, 0.0112 mols) was added to dimethylformamide (100 mL). This was followed by 3-(1,2,3,6-tetrahydropyridin-4-yl)-1*H*-indol (3) (2.44 g, 0.0123 mols, and triethylamine (5 mL). The resultant solution was heated at reflux for 18 h, after which it was cooled and the product crystalized out of solution. This was removed by filtration and washed with ethyl acetate to give 1.7 g of (4) in a 32% yield as a yellow solid. ^1^H NMR (DMSO) δ 11.17 (s, 1H), 8.03–7.87 (m, 5H), 7.50–7.40 (m, 6H), 7.23–7.07 (m, 2H), 6.21 (s, 1H), 3.34 (s, 2H), 2.95 (t, 2H), 2.79–2.74 (m, 4H), 2.60 (s, 2H); ^13^C NMR (DMSO) δ 201.02, 189.22, 167.13, 136.95, 134.68, 131.58, 129.66, 129.13, 127.21, 123.41, 120.10, 119.57, 119.22, 117.60, 107.17, 59.81, 52.77, 50.02, 41.18, and 32.59.

#### 4-(2-(4-(1*H*-indol-3-yl)-3,6-dihydropyridin-1(2*H*)-yl)ethyl)aniline (5)

2-(4-(2-(4-(1*H*-indol-3-yl)-3,6-dihydropyridin-1(2*H*)-yl)ethyl)phenyl)isoindoline-1,3-dione (**4**) (0.8 g, 0.0018 mols) was added to ethanol (100 mL) and hydrazine hydrate (2 mL) and gently warmed with stirring until a clear yellow solution was obtained. Stirring was continued at ambient temperature for 1 h then the solvent was removed under reduced pressure the residue was added to methylene chloride (100 mL) and stirred at ambient temperature for 18 h. Then the solution was filtered and washed with deionized water (100 mL), dried over magnesium sulfate filtered and evaporated to 0.4 g of product in a 70% yield, that appeared to be pure by NMR and was used in the next step without further purification. ^1^H NMR (CDCl_3_) δ 8.26 (s, 1H), 7.90 (d, 1H), 7.37 (d, 1H), 7.26–7.12 (m, 3H), 7.04 (d, 2H), 6.65 (d, 2H), 6.22 (s, 1H), 3.57 (brs, 2H), 3.22 (d, 2H), 2.84–2.50 (m, 8H); ^13^C NMR (CDCl_3_) δ 144.42, 136.75, 130.42, 129.71, 129.49, 125.25, 122.13, 121.19, 120.71, 119.97, 119.22, 118.07, 115.30, 111.27, 60.88, 53.15, 50.51, 33.10, and 29.15.

#### IDT785 (6)

4-(2-(4-(1*H*-indol-3-yl)-3,6-dihydropyridin-1(2*H*)-yl)ethyl)aniline (**5**) (0.05 g, 0.00016 mols) was added to biotin-PEG-SVA (0.1 g, 0.00002 mols) and this mixture was dissolved in methylene chloride. Then triethylamine (2 drops) was added and the solution was stirred at ambient temperature for 18 h. Finally, the solvent was removed under reduced pressure and the product was purified using size exclusion chromatography on Sephadex G10 resin to give 0.08 g of product IDT785 as a white powder.

### Materials in Biological Assays

DMEM FluoroBrite Live cell imaging medium, DMEM, fetal bovine serum, penicillin/streptomycin, Lipofectamine 3000, biotin-4-fluorescein (B4F) and QD655Sav (emission max at 655 nm) were purchased from Thermo Fisher Scientific. Poly-D-lysine hydrobromide (mol wt 70,000–150,000), bovine serum albumin (BSA), and paroxetine hydrochloride hemihydrate were purchased from Millipore Sigma. 35-mm uncoated No. 1.5 coverslip-bottomed dishes were purchased from MatTek. pEYFP-C1-hSERT was a gift from Harald Sitte (Addgene plasmid # 70103^1^).

### Cell Culture

HEK-293T cells were grown in a complete medium (DMEM with 2 mM glutamine, 10% FBS, 1% pen/strep) in a 37°C incubator with 5% CO_2_. Cells were seeded in 0.01 mg/mL poly-D-lysine-coated (1 h at 37°C) MatTek dishes for fluorescence imaging or in uncoated 24-well plates for IDT307 transport assay at an appropriate density to obtain a subconfluent monolayer and grown for 24 h in the complete growth medium. Then the cells were transiently transfected with 500 ng of the appropriate DNA per MatTek dish/well using Lipofectamine 3000 according to the manufacturer’s instructions.

### IDT307 Transport Assay

HEK-293T cells were seeded in 24-well plates, transfected with 500 ng hSERT pcDNA3 for 24 h, and then incubated with 10 μM IDT307 for 15 min in the presence of increasing concentrations of IDT785. Parallel wells preblocked with 10 μM paroxetine served as a negative control. IDT307 uptake was terminated by aspiration, and adherent HEK-293T cells were then trypsinized, transferred to microfuge tubes, centrifuged at 2500 rpm for 5 min, and resuspended in ice-cold DMEM FluoroBrite. A 100-μL aliquot of the cell suspension corresponding to a single well in a 24-well plate was added to the black-bottom 96-well plate. IDT307 fluorescence was collected on a BioTek Synergy H4 microplate reader using 480/20 nm excitation, 525/20 nm emission, and a gain of 100. Separately, a 50-μL aliquot of the cell suspension was then added to a coverslip and imaged at 20X (NA 0.4) on an inverted Axiovert 200M epifluorescence microscope using a standard GFP optical configuration.

### Molecular Docking

The three-dimensional structures of the docked compounds (paroxetine and IDT785-PEG_2_) were prepared in Chem3D 16.0. Docking analysis was performed using UCSF Chimera built-in AutoDock Vina to evaluate the hydrogen bond interaction and their binding affinities. The grid box was defined as 44 × 55 × 68 along the *x*, *y*, and *z* dimension, respectively, with the center *xyz* coordinates (126.823,130.899, and 110.557) at grid resolution of 1 Å for cryo-EM reconstructed ts2-inactive SERT–15B8 Fab–8B6 scFv complex with paroxetine in the outward-open conformation (PDB: 6DZW) to define the binding site for subsequent docking. Prior to docking, ts2-inactive SERT was separated from 15B8 Fab, 8B6 scFv, and paroxetine in UCSF Chimera. The following docking parameters were selected: number of binding modes – 10, exhaustiveness of search – 8, maximum energy difference (kcal/mol) – 3.

### Biotin-4-Fluorescein Quenching Assay

In black, flat-bottom 96-well plates, 2 nM QD655Sav was titrated in duplicate wells with biotin-4-fluorescein (B4F) from 3 to 40 nM. Samples were incubated for 1 h at room temp, in the dark, and then fluorescence was measured using a BioTek Synergy H4 microplate reader at 523/13.5 nm with excitation at 494/13.5 nm and an instrument gain of 75. QD655Sav was pre-blocked with either 10 μM IDT785 or 10 μM free biotin for 30 min and then titrated with B4F as previously described. All solutions were prepared in phosphate buffer saline (PBS) without Ca^2+^/Mg^2+^ (pH 7.4).

### QD655Sav Labeling

QD labeling was implemented via a two-step protocol (Chang et al., 2012a; Thal et al., 2020). After the cells were allowed 24 h to achieve transporter expression in MatTek dishes, labeling was carried out by first incubating the cells with IDT785 at 10 μM (or 500 nM for single QD tracking) for 10 min at 37°C. Following three washes with warm DMEM FluoroBrite, cells were then incubated with a 0.1 nM (or 0.05 nM for single QD tracking) QD655Sav diluted in warm DMEM FluoroBrite supplemented with 1% w/w BSA (labeling buffer) for 5 min at room temperature, washed three times with warm DMEM FluoroBrite, and used immediately for time-lapse image series acquisition. In negative control experiments, transfected HEK-293T cells were first blocked with 10 μM paroxetine for at least 10 min and then incubated with IDT785 in the presence of paroxetine.

### TIRF Microscopy and Colocalization Analysis

YFP-SERT/QD655 colocalization was imaged in the TIRF mode on a Nikon Eclipse Ti-E inverted microscope equipped with an Andor Zyla 4.2 PLUS sCMOS camera and viewed with an Apo TIRF 60×/1.49 NA oil objective. 488 nm excitation was sourced by a Nikon LU-NV laser unit. YFP-SERT emission was collected with a 525 ± 18 nm emission filter with an exposure time of 500 ms. QD655 signal was collected with a 655 ± 15 nm emission filter with an exposure time of 500 ms. Binary images of YFP-SERT were generated using adaptive thresholding algorithm in Matlab R2017b, the QD centroid positions for a given field of view were overlaid onto the binary YFP-SERT map, and the number/fraction of colocalized QDs for each dual channel field of view acquired were calculated.

### Spinning Disk Confocal Microscopy

Time-lapse image series were obtained on an inverted Nikon-Ti Eclipse microscope system equipped with the Yokogawa CSU-X1 spinning disk confocal scanner unit, a heated stage, a 60× oil-immersion Plan Apo 1.4 NA objective, and a back-illuminated sCMOS Prime95B camera. QDs were excited using a 405-nm solid state diode laser (23 mW), and QD655 emission was collected through the 641/150 emission filter. YFP-SERT molecules were excited using the 488-nm line (65 mW), and the YFP emission was collected using the 525/36 emission filter. Single QD tracking was performed at a scan rate of 10 Hz for 1 min at 60× or 100× (Apo TIRF Oil NA 1.49) magnification. SPT data were obtained within 20 min of the final wash step after QD labeling. Cross-section view images of hSERT-transfected HEK-293T were acquired at 100 ms exposure time with 3 × 3 stitching of 512 × pixel sections at 15% overlap.

### SIM Microscopy

SIM imaging was performed in single-plane 3D SIM mode on an inverted Nikon SIM microscope equipped Andor DU-897 EMCCD camera, a SR Apo TIRF 100× 1.49 NA oil-immersion objective, and 488 nm (74 mW) solid-state diode lasers used to excite YFP-SERT. Transfected HEK-293T were washed three times with warm DMEM FluoroBrite and imaged in warm DMEM FluoroBrite at room temperature.

### Trajectory Reconstruction and Analysis

Image analysis and trajectory construction were performed using ImageJ TrackMate plugin (Jaqaman et al., 2008; Tinevez et al., 2017). Intermittency of QD fluorescence was used to verify that single fluorophores were analyzed, and extracted trajectories were at least 50 frames in length to increase the robustness of statistical analysis (Chang and Rosenthal, 2011, 2013; Kovtun et al., 2020). Trajectories were considered continuous if a blinking QD was rediscovered within a 1 μm distance during the 10-frame time window. For each trajectory, mean square displacement (MSD), *r*^2^(*t*), was computed as follows:


(1) $$\left\langle r^{2}\left(n\delta t\right)\right\rangle =\frac{1}{N-n}\sum_{j=0}^{N-n-1}\left\{{\left[x\left(j\delta t+n\delta t\right)-x\left(j\delta t\right)\right]}^{2}+{\left[y\left(j\delta t+n\delta t\right)-y\left(j\delta t\right)\right]}^{2}\right\}$$

where δ*t* is the temporal resolution of the acquisition device, [*x*(*j*δ*t*), *x*(*j*δ*t*)] is the particle coordinate at t = *j*δ*t*, and N is the number of total frames recorded for an individual particle. Prior to MSD calculations, individual trajectories were reindexed with continuous time vectors to close the gaps caused by blinking and simplify MSD analysis. The diffusion coefficient *D*_*MLE*_ was determined through the use of a previously published Maximum Likelihood Estimation (MLE) theoretical framework to maximize performance in accurately calculating D (Michalet and Berglund, 2012). Trajectories with *D*_*MLE*_ < 5 × 10^–4^ μm^2^/s (equivalent to the 95th percentile value of *D*_*MLE*_ derived from the analysis of QDs immobilized on a glass coverslip) were considered immobilized. Additionally, a 2D kernel density was generated for each trajectory using a bivariate kernel density estimator with diagonal bandwidth matrix (Botev et al., 2010), and the vector field corresponding to the x and y components of instantaneous displacements was overlaid onto the 2D kernel density map. The angle θ between two successive vectors ***u*** and ***v*** was calculated based on the following formula:


(2) $$\cos\theta =\frac{\vec{u.}\vec{v}}{||\vec{u}||||\vec{v}||}$$

#### Relative Deviation Analysis to Classify Trajectory Motion Type

Independently of the motion mode, microscopic diffusion coefficient *D_2__–__4_* was determined by fitting the first 2–4 points of the MSD versus time curves with the equation:


(3) $${\left\langle r^{2}\left(t\right)\right\rangle }_{2-4}=4D_{2-4}t+offset$$

Four modes of motion are considered in order to describe the motional behavior of integral membrane proteins in the plasma membrane. These motional modes can be characterized on the basis of the plot of MSD versus time intervals:

(1) Stationary (immobilized) mode, in which a protein displays very little motion.
(2) Simple diffusion mode, in which a protein undergoes simple Brownian motion and its MSD-Δt plot is linear with a slope of 4D.
(3) Directed diffusion mode, in which a protein moves in a direction with a constant drift velocity with superimposed random diffusion with a diffusion coefficient D. In this case, the MSD-Δt plot is parabolic with a differential coefficient of 4D at time 0 (initial slope).
(4) Restricted diffusion mode, in which a protein undergoes Brownian motion within a limited area and cannot escape the area during the observation period (*0 ≤ x ≤ L_*x*_, 0 ≤ y ≤ L_*y*_*). This mode of motion is equivalent to free Brownian diffusion within an infinitely high square well potential. The slope of the MSD-Δt curve at time 0 is again 4D, and the MSD-Δt curve asymptotically approaches *L_*x*_^2^/6* and *L_*y*_^2^/6* in the x and y directions, respectively.

The MSD-Δt plot shows positive and negative deviations from a straight line with a slope of 4D (in the case of two-dimensional diffusion) for directed diffusion and restricted diffusion, respectively. Larger deviations indicate larger probabilities of non-Brownian diffusion. A parameter for the relative deviation, RD(N, n), is defined as:


(4) $$RD\left(N,n\right)=\frac{MSD\left(N,n\right)}{4D_{2-4}n\delta t}$$

where *MSD(N, n)* represents MSD determined at a time interval *n*δ*t* from a sequence of *N* video frames. *4D_2__–__4_n*δ*t* is the expected average value of MSD for particles undergoing simple diffusion with a diffusion coefficient of *D_2__–__4_* in two-dimensional space. In the case of simple diffusion, the average *RD(N, n)* should be 1 (Kusumi et al., 1993). Brownian trajectories with a diffusion coefficient of 0.1 μm^2^/s were generated by random walk simulations using experimentally relevant trajectory lengths (100, 200, 300, 400, 500, and 600 steps) to establish the effective cutoff values of RD at 25 frames (Kovtun et al., 2019). RD values within the 2.5th–97.5th percentile range were taken to represent statistical variations in Brownian motion, and those outside of the range taken as restricted diffusion. A linear least-squares fit to the 2.5th percentile points defined the lower boundary for free diffusion, with trajectories having RD(N,25) below this line classified as restricted. Since YFP-SERT at the plasma membrane should not be actively transported by intracellular processes, directed diffusion represented a small fraction of all trajectories and trajectories with RD(N,25) above the polynomial fit of the 97.5th percentile points were classified as free (Crane and Verkman, 2008).

## Results

### IDT785 Inhibition of hSERT-mediated IDT307 Uptake

**FIGURE 2:**
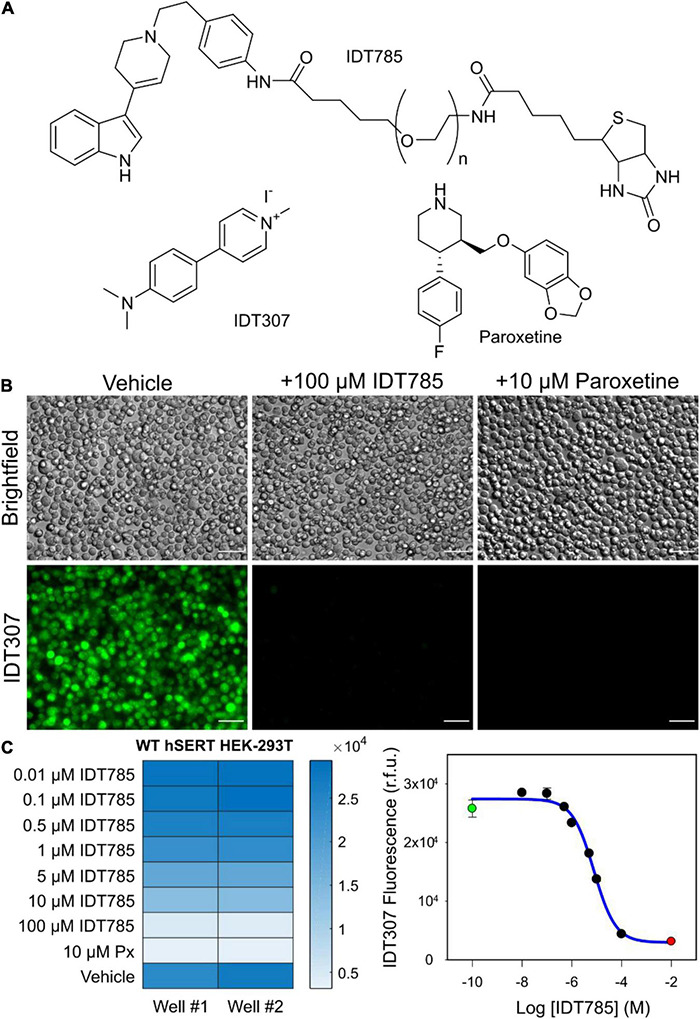
IDT785 inhibits hSERT-mediated IDT307 transport in a dose-dependent manner. **(A)** Structures of compounds used in the IDT307 transport assay are shown. **(B)** Untreated hSERT-expressing HEK-293T cells demonstrated characteristic IDT307 (green) intracellular accumulation, whereas cells pretreated with either 100 μM IDT785 or 10 μM paroxetine showed significantly reduced IDT307 uptake. Scale bar: 50 μm. **(C)** The heat map of microplate reader fluorescence measurements of hSERT-HEK-293T cell suspensions treated with varying IDT785 doses is shown. The relative IC_50_ value of IDT785 for inhibition of IDT307 transport was determined to be 7.2 ± 0.3 μM (mean ± s.e.m.), *n* = 3. IC_50_ was determined by using non-linear regression curve fit for sigmoidal dose response (one-site competition) in Sigma Plot 12. Representative inhibition curve from single experiment is shown here. Data points from unblocked cells (green) and paroxetine-blocked cells (red) were used to establish the top and bottom plateau, respectively.

To determine if IDT785 inhibited hSERT uptake function, a fluorescence transport assay was carried out using IDT307 (APP+), a fluorescent substrate for monoamine transporters (Singh et al., 2012; Solis et al., 2012; Beikmann et al., 2013). IDT307 is a twisted intramolecular charge transfer compound (TICT) and only fluoresces once it is transported inside the cell, where it assumes the active (emissive) coplanar conformation by binding to intracellular biomolecules, with a particular preference for nucleic acids enriched in mitochondria and nucleoli (Figure 2A; Solis et al., 2012). As expected, hSERT-expressing HEK-293T cells incubated with 10 μM IDT307 for 15 min exhibited characteristic intracellular accumulation of IDT307 most pronounced in the mitochondria and nucleoli, whereas cells pretreated with excess IDT785 (100 μM) and 10 μM paroxetine [SERT binding affinity of ∼70 pM according to Cool et al. (1990)] showed significantly reduced intracellular IDT307 accumulation. Next, the relative half-maximal inhibitory concentration (IC_50_) of IDT785 for inhibition of IDT307 uptake was determined using the BioTek Synergy H4 microplate reader. Briefly, IDT307 uptake was measured for hSERT-expressing HEK-293T cells incubated in the presence of increasing IDT785 dose or paroxetine (negative control) in duplicate wells (Figure 2C). Incubation of hSERT-expressing cells with IDT785 resulted in a concentration-dependent IDT307 uptake inhibition, and the fitted dose-response curve appeared sigmoidal in each independent experiment. IDT307 accumulation in cells treated with 100 μM IDT785 did not reach complete inhibition as defined by paroxetine-treated cells; thus, IDT307 accumulation in paroxetine-treated cells was used as the complete inhibition data point (Weimer et al., 2012). The IC_50_ value was determined to be 7.2 ± 0.3 μM (mean ± s.e.m.) on the basis of three independent experiments, with a representative dose-response curve from a single experiment shown in Figure 2C. Our data suggest that although the attachment of spacer-PEG-biotin to the tetrahydropyridine nitrogen atom of the parent drug resulted in a relative reduction of the binding affinity [IC_50_ of 690 ± 270 nM for 3-(1-methyl-1,2,3,6-tetrahydropyridin-4-yl)-1H-indole; Deskus et al., 2007], IDT785 still possessed biological activity and inhibited hSERT transport function in a dose-dependent manner.

### IDT785 Inhibition of QD655Sav-mediated Biotin-4-fluorescein (B4F) Quenching

**FIGURE 3:**
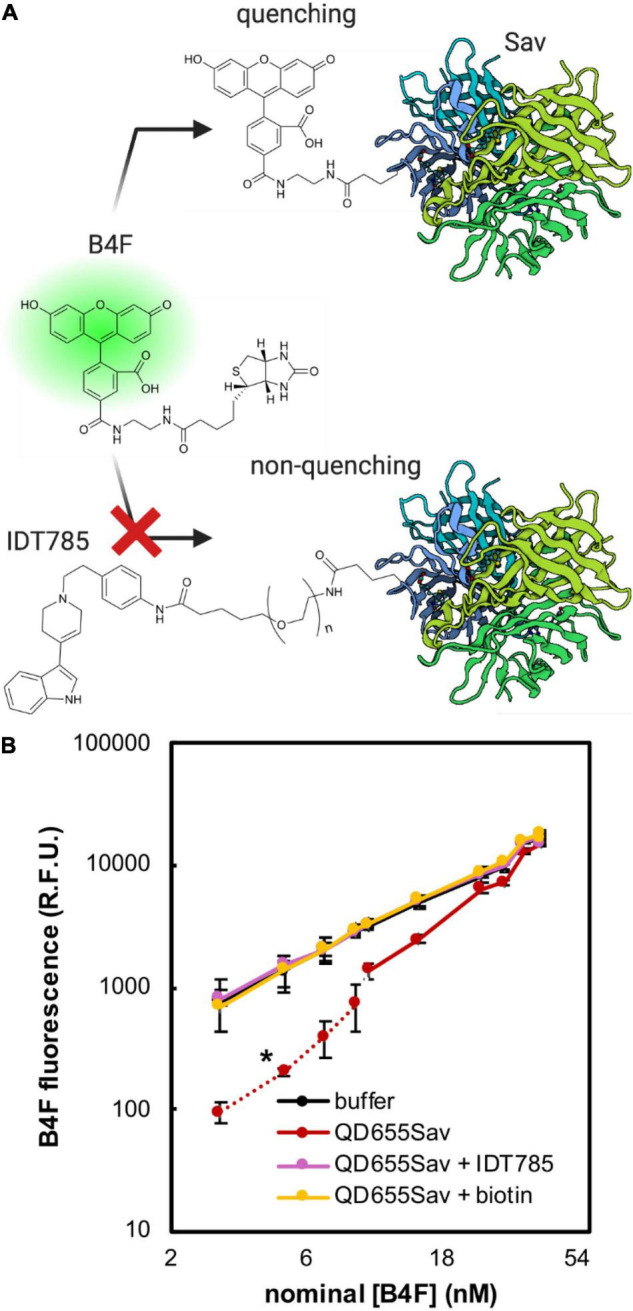
Biotin-4-fluorescein (B4F) quenching assay to validate IDT785 biotin moiety availability. **(A)** Illustration of B4F quenching action. B4F fluorescence is quenched when it binds to streptavidin (Sav) at the QD surface. Pre-blocking QD-bound streptavidin with biotinylated IDT785 prevents B4F from binding, leading to retention of B4F fluorescence. Streptavidin structure was based on the RCSB PDB entry 1SWE. **(B)** Averaged titration profiles of 2 nM QD655Sav with B4F. Dashed red line indicates [B4F] range of strong quenching regime. Once all active sites are filled, fluorescence increases linearly similar to that of buffer (statistical comparison at [B4F] = 3, 5, 7, and 9 nM resulted in *p < 0.05, unpaired Student’s *t*-test; *n* = 4 fluorescence intensity measurements per data point from 2 independent titrations). Negative controls: (1) QD655Sav pre-blocked with 10 μM IDT785, (2) QD655Sav pre-blocked with 10 μM free biotin, and (3) buffer only without QD655Sav.

To determine if the biotin moiety of IDT785 was available for binding to the Sav active site on QD655Sav, a biotin-4-fluorescein (B4F) quenching assay was implemented, previously used to quantify the amount of Sav present on the surface of QDs (Mittal and Bruchez, 2011; Lippert et al., 2016). B4F fluorescence quenching is facilitated through charge transfer from fluorescein to the outward-facing Sav residues, only occurring when they are brought into close proximity by biotin binding (Figure 3A). Thus, Sav-mediated B4F quenching can be used in a competition assay format to validate IDT785 biotin binding to QD655Sav. Two fluorescence regimes exist for B4F quenching (Mittal and Bruchez, 2011). The first occurs at low nM B4F concentrations, where B4F quantitatively binds to QD655Sav and B4F fluorescence is significantly reduced. At 4:1 B4F:Sav stoichiometric ration, B4F fluorescence is reduced by up to 88% (Mittal and Bruchez, 2011). Once all biotin-binding sites are occupied with B4F, a second linear concentration-dependent regime is typically observed (Figure 3B). As expected, QD655Sav pre-blocked with either 10 μM free biotin or 10 μM biotinylated IDT785 exhibited a linear fluorescence regime at increasing B4F concentrations identical to B4F titration in buffer only (Figure 3B), indicating that IDT785 competitively inhibited B4F binding. These data confirmed that the PEG moiety did not interfere with IDT785 biotin terminus binding to Sav presented at the QD surface.

### IDT785 Docking to ts2-inactive Outward-Facing SERT

**FIGURE 4:**
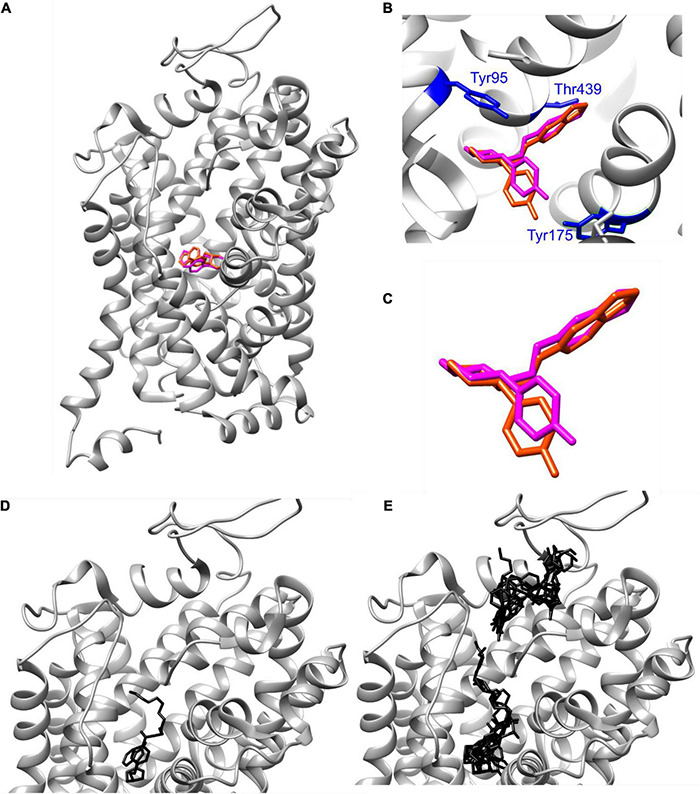
AutoDock Vina docking of paroxetine and IDT785 drug moiety to hSERT. **(A–C)** Poses of paroxetine (magenta) docked via AutoDock Vina into ts2-inactive SERT are compared to the published cryo-EM SERT structure in complex with paroxetine (dark orange) (PDB: 6DZW). **(D)** Best-scoring (–9.8 kcal/mol) pose of PEG_2_-spacer-IDT785 docked into ts2-inactive SERT is shown in black, occupying the central binding site with the PEG terminus extending toward the extracellular space. **(E)** Second binding site (range: [–7.6, –7.1] kcal/mol) for PEG_2_-spacer-IDT785 was detected within the extracellular region of SERT.

Structural basis for binding of the diverse family of SSRIs to SERT has been under increasing scrutiny. Elegant engineering of thermostable SERT variants has resulted in solved X-ray crystal structures of SERT complexed with various antidepressants, and recent advances in single-particle cryo-EM have shed further light on molecular mechanisms of SERT inhibition by SSRIs (Coleman et al., 2016, 2019, 2020; Coleman and Gouaux, 2018). To gain insight into the interaction of IDT785 drug moiety with SERT, molecular docking was performed for a short version of IDT785 featuring the parent drug, spacer, and the PEG_2_ terminus as well as paroxetine (control docking) into the outward-facing ts2-inactive SERT structure (PDB: 6DZW), which was previously solved in complex with paroxetine bound to the orthosteric site using single-particle cryo-EM (Coleman et al., 2019). Molecular docking was carried out using UCSF Chimera 1.4 with AutoDock Vina 1.1.2, and the docked complex for each compound was analyzed on the basis of binding affinity (score in kcal/mol) and hydrogen bond interactions with the amino acid residues within SERT (Figure 4). Best-scoring paroxetine binding pose (Figures 4A–C) had the binding affinity of −9.8 kcal/mol and docked into the central binding site in good agreement with the reported SERT-bound paroxetine pose shown in purple. Root-mean-square deviation (RMSD) between the reported paroxetine pose and the docked pose in our analysis was calculated to be 0.789Å using open-source DockRMSD (Bell and Zhang, 2019). Hydrogen bond analysis revealed an interaction between the nitrogen atom and Asp98 residue with a distance of 2.327Å as well as Tyr95 with a distance of 2.323Å. Additionally, the benzodioxol oxygen of paroxetine was found to interact with Thr349 with a distance of 3.229Å. Best-scoring IDT785-spacer-PEG_2_ pose (Figure 4D) had the binding affinity of −9.4 kcal/mol, with the drug moiety occupying the central binding site and the PEG chain extending into the extracellular vestibule. Hydrogen bond analysis revealed amide bond nitrogen interaction with both oxygen atoms (2.259 and 2.299Å) of Glu493, an extracellular gating residue. Figure 4E shows all detected IDT785-spacer-PEG_2_ conformations docked into SERT structure, with a cluster of lower-scoring poses (binding scores between −7.6 and −7.1 kcal/mol) were located between EL2 and EL4 in the extracellular portion of the transporter. Overall, it appears that IDT785 is able to access the central binding site previously shown to be occupied by paroxetine, although it is unclear whether the addition of a PEG chain impairs the ability of IDT785 to penetrate past the extracellular gate into the orthosteric site.

### IDT785-QD Binding to Surface hSERT and YFP-SERT Proteins

**FIGURE 5:**
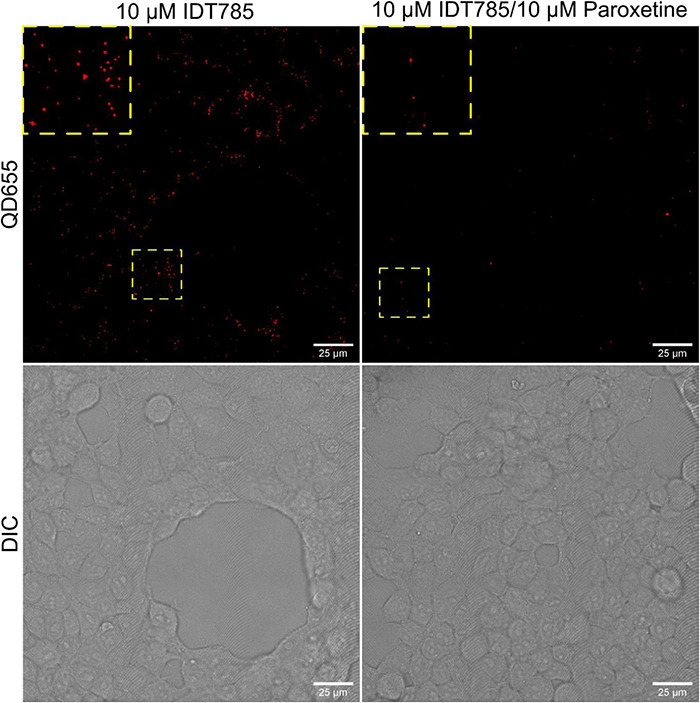
IDT785 enables membrane hSERT labeling with streptavidin-conjugated QDs. Stitched (3 × 3) field of views of hSERT-HEK-293T cells sequentially labeled with 10 μM IDT785 and 0.1 nM QD655Sav (red) in the absence or presence of 10 μM paroxetine were acquired via spinning disk confocal microscope at the depth of ∼5 μm away from the coverslip-membrane interface. Punctate labeling pattern that is reduced in the presence of paroxetine is indicative of specific QD labeling of surface hSERT proteins. Yellow boxes indicate areas shown in the inset squares at digital zoom of ∼2. Scale bar: 25 μm.

**FIGURE 6:**
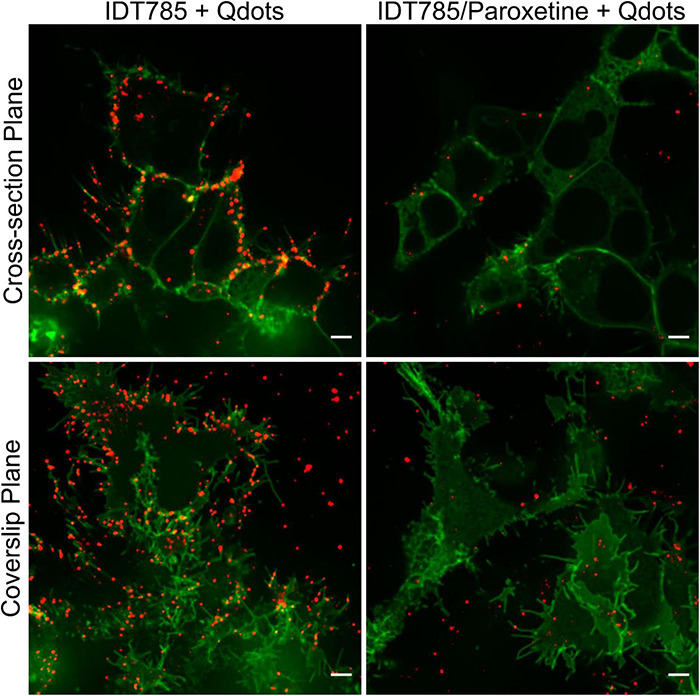
IDT785 enables membrane YFP-SERT labeling with streptavidin-conjugated QDs. Cross-section (∼5 μm away from the coverslip) and coverslip images of YFP-SERT-expressing (green) HEK-293T cells sequentially labeled with 10 μM IDT785 and 0.1 nM QD655Sav in the absence or presence of 10 μM paroxetine were acquired via spinning disk confocal microscope. Characteristic membrane-associated QD labeling pattern (red) that is reduced in the presence of paroxetine is readily apparent. Note a small fraction of QDs non-specifically bound to the coverslip outside YFP-SERT membrane regions. Scale bar: 5 μm.

**FIGURE 7:**
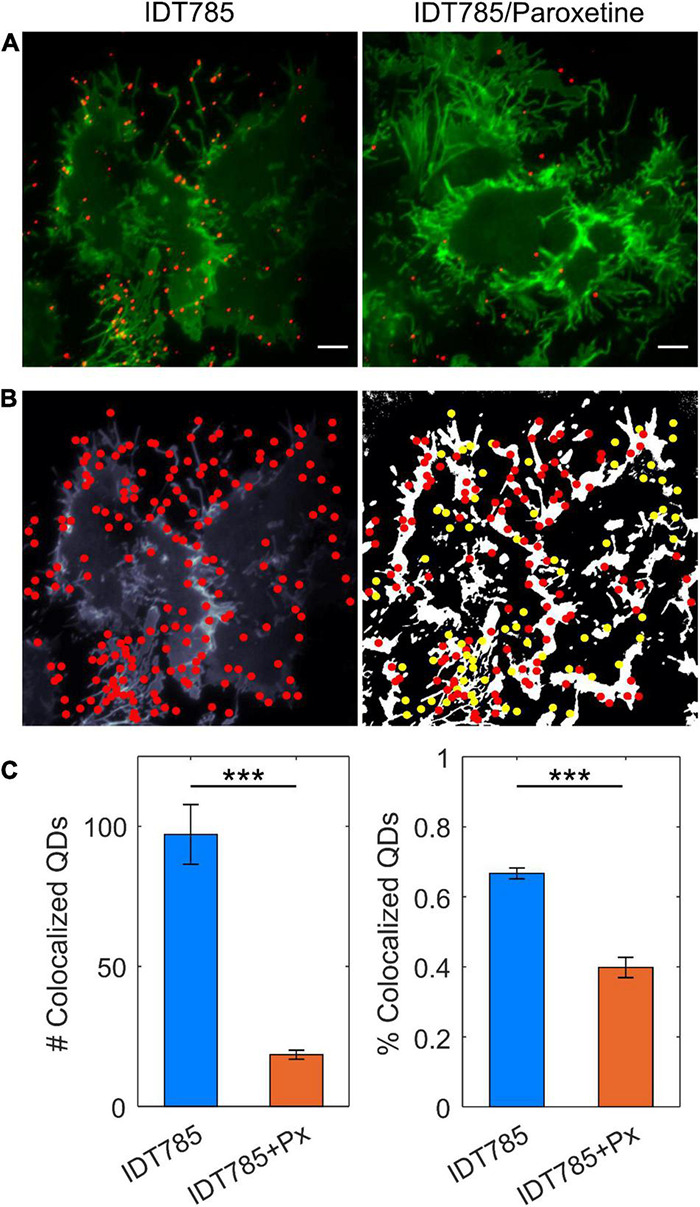
Quantitation of specific QD labeling of YFP-SERT at the membrane-coverslip interface. **(A)** Representative TIRF images of YFP-SERT-HEK-293T cells sequentially labeled with 10 μM IDT785 and 0.1 nM QD655Sav in the absence or presence of 10 μM paroxetine. Scale bar: 5 μm. **(B)** Center positions of individual QDs were estimated with a 2D Gaussian fitting algorithm and overlaid onto binarized maps of YFP-SERT distribution in HEK-293T cells. Colocalization (red) and non-colocalization (yellow) of QDs with YFP-SERT were determined for a given 512 × 512 pixel field of view. **(C)** Number and fraction of colocalized QDs were determined in the absence (*n* = 17 fields of view; blue) and presence (*n* = 23 fields of view; orange) of paroxetine. Number of colocalized QDs: 97 ± 10 for IDT785 vs. 18 ± 2 for IDT785+Px; data presented as mean ± s.e.m.; ***p < 0.0001, unpaired Student’s *t*-test. Fraction of colocalized QDs: 67 ± 2% for IDT785 vs. 40 ± 3% for IDT785+Px; data presented as mean ± s.e.m.; ***p < 0.0001, unpaired Student’s *t*-test.

After biological activity had been confirmed for both the drug end and the biotin end of IDT785, its ability to enable QD labeling of surface hSERT proteins transiently expressed in HEK-293T cells was evaluated. In a two-step labeling protocol, the cells were labeled first with 10 μM IDT785 for 10 min and then with 0.1 nM QD655Sav for 5 min. QD-labeled cells were imaged on the spinning disk confocal microscope immediately after the last wash step. Stitching 3 × 3 images with 15% overlap at the edges allowed us to capture a large field of view (FOV) of 298 × 298 μm^2^ covering tens of cells (Figure 5). hSERT-HEK-293T cells preblocked with 10 μM paroxetine and then incubated with 10 μM IDT785 in the presence of 10 μM paroxetine were used as a negative control. A characteristic pattern of QD labeling that appeared to be associated with the membrane edges in the cross-section focal plane was apparent. Cells blocked with paroxetine had considerably fewer bound QD spots indicative of specific hSERT targeting. To obtain a more quantitative measure of QD labeling specificity, HEK-293T cells were transiently transfected with wild-type hSERT tagged with a yellow fluorescent protein at the intracellular N terminus (YFP-SERT) (Sucic et al., 2010; Montgomery et al., 2014). YFP-SERT-expressing cells were labeled with 10 μM IDT785 and 0.1 nM QD655Sav in a two-step protocol, with paroxetine-blocked cells serving as a negative control. Figure 6 shows representative images of the extent of QD labeling of membrane YFP-SERT at the membrane-coverslip interface as well as a few microns away from the coverslip, so that QDs non-specifically adsorbed to the coverslip are omitted (cross-section view). Visual inspection of paroxetine-blocked YFP-SERT-transfected cells revealed considerably lower extent of membrane QD labeling, in agreement with the previous result of untagged wild-type hSERT labeling. However, a fraction of QDs was non-specifically associated with both the plasma membrane and the coverslip surface in paroxetine-blocked cells. Since YFP-SERT appeared to be enriched in the membrane edges and protrusions at the membrane-coverslip interface (Figures 6, 7A; Supplementary Figure 1), images of YFP-SERT distribution acquired in the TIRF mode were binarized using adaptive thresholding in Matlab and the colocalization of individual QD centroid positions with YFP-SERT for each acquired field of view (FOV) was determined (Figure 7B). The total number of colocalized QDs and the relative fraction of colocalized QDs per acquired FOV were significantly greater for YFP-SERT-expressing cells labeled with IDT785 and QD655Sav in the absence of paroxetine block (Figure 7C), thus confirming labeling specificity.

### IDT785-enabled Single QD Tracking of YFP-SERT

**FIGURE 8:**
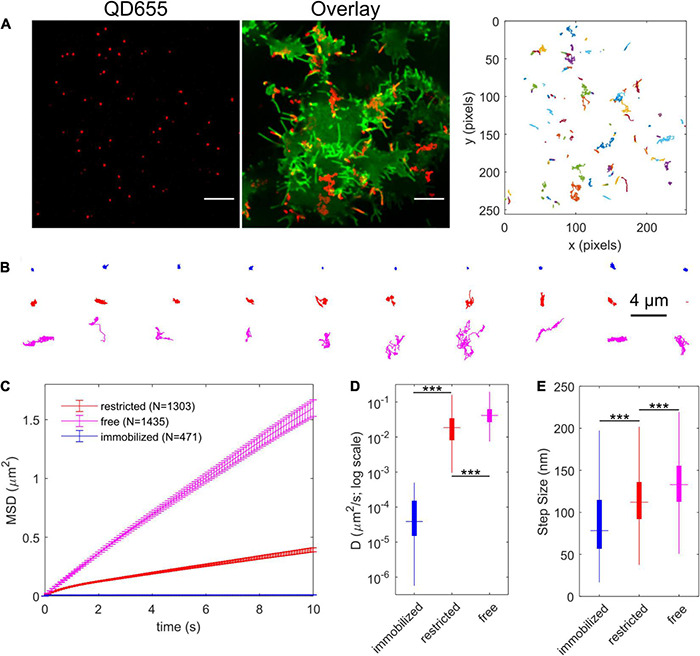
IDT785-QD labeling enables single QD tracking of YFP-SERT in HEK-293T cells. **(A)** Representative spinning disk confocal images show YFP-SERT localization pattern and the corresponding QD labeling. Note the relative increase in YFP-SERT intensity in the protrusions. Scale bar: 10 μm. Middle panel shows maximum intensity projection of QD signal over time (600 frames recorded at 10 Hz) in red overlaid onto YFP-SERT image. Right panel shows reconstructed trajectories corresponding to the tracks apparent in the middle panel. A range of diffusive behavior is readily apparent. **(B)** Relative Deviation (RD) analysis identifies three distinct types of diffusion exhibited by YFP-SERT-QD trajectories. Ten representative trajectories are shown for each diffusion type – immobilized (blue; D < 5 × 10^–4^ μm^2^/s), restricted (red), and free or Brownian (magenta). **(C)** Time-dependent averaged MSD curves are shown for YFP-SERT-QD trajectories that are immobilized (471 tracks from 3 independent experiments; 15% of total), undergo restricted diffusion (1303 tracks from 3 independent experiments; 40%), and undergo free diffusion (1435 tracks from 3 independent experiments; 45%). MSD curves include weighted standard deviation error bars. **(D)** Box plot shows the diffusion coefficient distribution for each diffusion type trajectories. The median value is shown as the colored horizontal line in the box, the 25–75% IQR interval corresponds to the length of the colored box, and whiskers extend to 5 and 95% percentile values. The distributions were compared using the Mann-Whitney *U* test (***p < 0.001, immobilized tracks: D_median_ = 3.9 × 10^–5^ μm^2^/s, IQR = [1.5 × 10^–5^, 1.5 × 10^–4^]; restricted diffusion: D_median_ = 0.019 μm^2^/s, IQR = [0.008, 0.034]; free diffusion: D_median_ = 0.041 μm^2^/s, IQR = [0.027, 0.062]). **(E)** Box plot shows the instantaneous step size (per Δt = 100 ms) distribution for trajectories of each diffusion type. The median value is shown as the colored horizontal line in the box, the 25–75% IQR interval corresponds to the length of the colored box, and whiskers extend to 5 and 95% percentile values. The distributions were compared using the Mann-Whitney *U* test (***p < 0.001, immobilized tracks: step size_median_ = 78 nm, IQR = [57, 115]; restricted diffusion: step size_median_ = 112 nm, IQR = [92, 136]; free diffusion: step size_median_ = 133 nm, IQR = [113, 155]).

To track QD-bound YFP-SERT, transfected HEK-293T cells were labeled stepwise with 500 nM IDT785 for 10 min and 0.05 nM QD655Sav for 5 min and imaged for 1 min at 100 ms exposure time (10 Hz) to record the lateral movement of single QDs. Specificity of QD labeling was confirmed at these conditions by imaging YFP-DAT-transfected HEK-293T cells in parallel (Supplementary Figure 2). Representative maximum intensity projection (MIP) image of a 1-min sequence of QD movement overlaid onto YFP-SERT image and corresponding reconstructed QD trajectories are displayed in Figure 8A. A range of diffusive behavior was readily apparent from the reconstructed QD trajectory shape. Next, a Relative Deviation (RD) analysis was implemented to classify experimental QD trajectories as freely diffusing or restricted on the basis of mean square displacement (MSD)-Δt curve deviation compared to simulated trajectories undergoing simple Brownian motion (Kusumi et al., 1993; Kovtun et al., 2019). Additionally, QD trajectories diffusing at a rate slower than *D*_cutoff_ = 5 × 10^–4^ μm^2^/s (95th percentile of diffusion coefficient distribution of QDs dropcast onto a coverslip) were categorized as immobilized (Dahan et al., 2003; Kovtun et al., 2019). Figure 8B displays 10 randomly selected trajectories in each category of diffusion type. In total, 40% of QD trajectories (*n* = 1303) were classified as restricted diffusion, 45% (*n* = 1435) of QD trajectories were determined to exhibit simple Brownian diffusion, and 15% (*n* = 471) of QD trajectories were considered to be immobile, comprised of both immobile QD-bound YFP-SERT and QDs non-specifically adsorbed to the coverslip. Time-averaged ensemble MSD-Δt curves of immobilized and restricted QD trajectories exhibited characteristic downward curvature indicative of confined diffusion, whereas MSD-Δt curve for freely diffusing QDs was characterized by linear increase, as expected for a case of simple Brownian diffusion (Figure 8C). Analysis of individual trajectories revealed that Brownian trajectories were diffusing laterally at the rate of 0.04 μm^2^/s (median *D*_*MLE*_) and instantaneous velocity of ∼1.3 μm/s, approximately twice the diffusion rate of trajectories classified as restricted (*D_*MLE*_* = 0.019 μm^2^/s) and ∼100 × faster than the diffusion rate of immobilized QD pool (Figures 8D,E). Overall, IDT785 enabled single QD tracking of YFP-SERT surface dynamics in transfected HEK-293T cells and subsequent classification of distinct motion modes exhibited by individual trajectories at the nanoscale.

### Protrusion Localization and Membrane Dynamics of YFP-SERT

**FIGURE 9:**
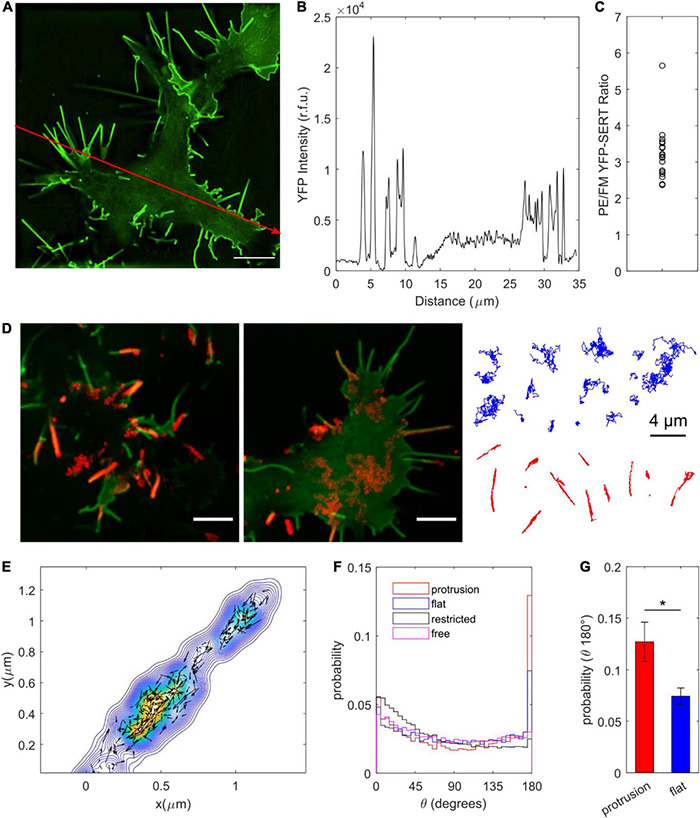
Surface dynamics of YFP-SERT pool localized to the membrane protrusions. **(A)** A representative SIM image demonstrates YFP-SERT localization pattern in transiently transfected HEK-293T cells. Scale bar: 5 μm. **(B)** An intensity line plot corresponding to the red line drawn in **(A)** indicates the presence of high-density YFP-SERT cellular regions localized to the membrane protrusions and membrane edges. **(C)** A scatter plot of the ratio of YFP-SERT intensity in protrusions/edges (PE) vs. flat membrane zones (FM) reveals a ∼300% enrichment of YFP-SERT in PE membrane regions (mean ± s.e.m., PE/FM YFP intensity: 3.2 ± 0.2; data are based on 19 1024 × 1024 reconstructed fields of view acquired in three independent experiments). **(D)** Two representative images demonstrate SERT-QD diffusion in distinct cellular regions – flat membrane (FM) zones and membrane protrusions. Maximum intensity projections of the QD channel of the time-lapse image series (600 frames at 10 Hz, 100×) were overlaid onto YFP-SERT images. Scale bar: 5 μm. Representative trajectories are shown for each membrane region of interest (FM in blue, protrusions in red). **(E)** A representative color-coded bivariate 2D kernel density contour plot corresponds to a single YFP-SERT-QD protrusion trajectory and includes scaled velocity vectors for single-step instantaneous displacements. Bidirectional motion along the protrusion is readily apparent. **(F)** Probability distributions for the angle θ between two successive displacement vectors are displayed for QD trajectories localized to protrusions (*n* = 64,062 steps) and flat membrane regions (*n* = 66,072 steps), as well as previously classified trajectories undergoing restricted diffusion (*n* = 1,045,575 steps) and Brownian diffusion (*n* = 855,570 steps). θ = 0 degrees corresponds to motion reversal, whereas θ = 180 degrees corresponds to continued progress in a given direction, indicative of unimpeded diffusion. **(G)** Protrusion trajectories (*n* = 110; red) exhibited greater probability of the calculated θ within the 175–180 degree range compared to flat membrane region trajectories (*n* = 113; blue) (mean ± s.e.m.; 0.12 ± 0.2 vs. 0.07 ± 0.01; *p < 0.05, unpaired Student’s *t*-test).

TIRF imaging to validate ITD785 revealed that YFP-tagged hSERT was enriched in the membrane edges and thin protrusions. Interestingly, converging lines of evidence indicate that neurotransmitter transporters undergo highly specialized sorting, targeting, and retention, which results in a distinct (heterogeneous) surface transporter distribution both *in vitro* and *vivo* (Radian et al., 1990; Minelli et al., 1995; Nirenberg et al., 1997; Zhou et al., 1998; Tao-Cheng and Zhou, 1999; Miner et al., 2000; Steiner et al., 2008; Rao et al., 2012; Montgomery et al., 2014; Block et al., 2015; Caltagarone et al., 2015; Ma et al., 2017; Kaya et al., 2018). In particular, SERT was shown to primarily exist outside the synaptic junctions along the raphe neuron axonal membrane in a highly polarized pattern (Zhou et al., 1998; Tao-Cheng and Zhou, 1999). Catalytically active SERT was reported to preferentially localize to the actin-rich focal adhesions of resting human platelets (Steiner et al., 2008). Moreover, export of both endogenous SERT and heterologously expressed YFP-SERT from the endoplasmic reticulum (ER) is directed toward axonal tracts of cultured rat dorsal raphe neurons (Montgomery et al., 2014). To quantify the extent of YFP-SERT preferential localization to the edge and protrusion (PE) regions, super-resolved images of YFP-SERT-expressing HEK-293T cells were captured on a structured illumination microscope in a 3D mode (Figure 9A). Pronounced peaks in the line intensity profile shown in Figure 9B indicate a large increase of the YFP-SERT density in PE membrane regions compared to the flat membrane (FM) zones. The average YFP intensity ratio for PE:FM regions was calculated to be equivalent to approximately 300% enrichment of YFP-SERT in the PE regions, consistent with visually apparent YFP-SERT polarized distribution (Figure 9C). Next, at least 100 QD trajectories were manually selected for protrusion- and FM-localized YFP-SERT from time-lapse videos acquired at 100 × magnification for 1 min at 10 Hz for subsequent MSD and diffusion analysis (Figure 9D). Motion of QD-labeled YFP-SERT along the protrusions was Brownian in nature, as evidenced by the linear MSD-Δt curve and the median diffusion coefficient of 0.041 μm^2^/s, statistically identical to that of trajectories classified as freely diffusing in the previous section (Supplementary Figure 3). Additionally, 2D kernel density contour maps were constructed for individual protrusion trajectories and visualized with overlaid instantaneous velocity vectors in Figure 9E. QD-bound YFP-SERT motion along the protrusion was bidirectional in nature, with frequent direction switching along the major protrusion axis. According to Lam et al. (2019), the higher probability of small angle θ between successive frame-to-frame steps indicates frequent occurrence of directional switching that may be suggestive of diffusion being impeded by submicron-sized compartments. Therefore, the angle θ was calculated for QD-bound YFP-SERT trajectories, with θ = 0° corresponding to direction reversal and θ = 180° indicating unimpeded forward motion (Figures 9F,G). The probability of θ in the range of 175–180° for protrusion-localized trajectories was significantly greater than θ in the identical range for FM region-localized trajectories and θ in the range of 0–5° for protrusion-localized trajectories, corroborating that QD-bound YFP-SERT complexes exhibited largely unimpeded diffusion within membrane protrusions. Together, these data further demonstrate that IDT785 readily enables QD-based characterization of YFP-SERT diffusion patterns at the nanoscale.

## Discussion

Over the past two decades, ultrastructural electron microscopy and immunofluorescence approaches have revealed a unique pattern of SERT distribution in the plasma membrane in both brain- and periphery-derived cultures (Zhou et al., 1998; Tao-Cheng and Zhou, 1999; Miner et al., 2000; Steiner et al., 2008; Montgomery et al., 2014). The molecular mechanisms that enable optimal SERT positioning for rapid clearance of extracellular serotonin remain largely unknown, and trafficking-dependent regulation of SERT function has been an area of active investigation. The study of SERT trafficking in live cell cultures has been significantly limited by the lack of suitable antibodies that target the extracellular domain of the transporter. To combat this challenge, the Rosenthal laboratory pioneered the use of small-molecule neurotransmitter or antagonist derivatives to enable real-time monitoring of SERT trafficking at the cell surface (Rosenthal et al., 2002; Tomlinson et al., 2011; Chang et al., 2012b). Our current probe design features an attachment of a biotinylated, PEGylated spacer to a transporter-specific antagonist, which can then be readily detected by streptavidin-conjugated QDs and facilitate single QD tracking of transporter membrane dynamics. In this approach, the point of spacer attachment must be carefully chosen to preserve parent antagonist binding affinity for SERT; moreover, the spacer identity is critically important for stabilizing the antidepressant-transporter interaction as well as minimizing non-specific ligand binding to the plasma membrane or coverslip. In particular, our recent report demonstrated that replacing a flexible alkyl (undecyl) spacer with a more rigid, electron-rich, less hydrophobic phenylethyl spacer in the structure of biotinylated D2 dopamine receptor ligand significantly reduced non-specific ligand binding to the coverslip surface (Tomlinson et al., 2019). Thus, our goal in the current study was to incorporate the phenylethyl spacer into the SERT ligand design and determine its effect on parent drug affinity, ligand binding specificity, and consequently the ability to monitor surface SERT dynamics with ligand-conjugated QDs.

Modification of the pyridinyl nitrogen in the parent drug 3-(1,2,3,6-tetrahydropyridin-4-yl)-1H-indole was found to be relatively well-tolerated (Deskus et al., 2007; Tomlinson et al., 2011). The methyl-substituted analog 3-(1-methyl-1,2,3,6-tetrahydropyridin-4-yl)-1H-indole was determined to inhibit [^3^H]citalopram binding to hSERT in the membrane homogenates of stably transfected HEK-293 cells with the IC_50_ value of 690 ± 270 nM (Deskus et al., 2007). Our concerns were that the steric constraints of the extracellular SERT vestibule might not support the bulkier phenylethyl spacer extending into the extracellular space and slightly lower hydrophobicity of the aromatic ring compared to an alkyl chain might reduce binding stability. To measure the binding affinity of the biotinylated, PEGylated 3-(1-phenethyl-1,2,3,6-tetrahydropyridin-4-yl)-1H-indole (IDT785), an IDT307 (APP+) fluorescent substrate transport assay was employed. IDT307 is actively transported across the membrane via monoamine neurotransmitter transporters (SERT, DAT, NET) as well as organic cation transporters 1–3 (OCT 1–3) and permits rapid characterization of transporter-inhibitor interaction in a 96-well plate format (Duan et al., 2015). The IC_50_ values determined by this method show minimal plate-to-plate or day-to-day variability. hSERT-mediated uptake in transiently transfected HEK-293T cells was effectively inhibited by excess (100 μM) IDT785 and 10 μM paroxetine (negative control) (Figure 2), indicating that the phenylethyl spacer did not adversely affect the biological activity of 3-(1,2,3,6-tetrahydropyridin-4-yl)-1H-indole. The IC_50_ of IDT785 was determined to be 7.2 ± 0.3 μM using IDT307-based inhibition assay, comparable to our previously reported compounds based on 3-(1,2,3,6-tetrahydropyridin-4-yl)-1H-indole featuring the undecyl linker (Tomlinson et al., 2011). Admittedly, the IC_50_ of the final biotinylated ligand is quite high and an order of magnitude larger than the IC_50_ of the drug intermediate before the PEGylation step (Supplementary Figure 4). Our next goal is to achieve sub-μM affinity before we proceed with SERT tracking experiments in neuronal cultures or intact tissue. At high ligand doses, one might anticipate several potential undesirable off-target effects, including activation of serotonin receptors [the parent drug of IDT785 is a derivative of RU-24969, a preferential 5-HT_1B_ agonist, with a K_i_ of 0.38 nM, which also displays appreciable affinity for the 5-HT_1A_ receptor (K_i_ = 2.5 nM) (Aronsen et al., 2014)] or incorporation of the lipophilic moiety of IDT785 into membrane rafts (Erb et al., 2016). These off-target effects can in turn influence SERT surface state by inducing changes in membrane polarization through the activation of membrane ion channels, G protein-mediated stimulation of signaling pathways that control SERT trafficking/phosphorylation/palmitoylation, or local disruption of membrane rafts that are known to stabilize transport-willing SERT conformation. Next, a B4F quenching assay was performed to rule out the possibility (although unlikely) that the PEG chain sterically hindered the availability of the IDT785 biotin end for binding to Sav conjugated to QDs. Quenching of B4F fluorescence upon free Sav or SavQD binding is a well-documented phenomenon, with a nearly 90% B4F fluorescence reduction achieved at B4F:Sav molar ratios ≤ 4:1 (Mittal and Bruchez, 2011; Lippert et al., 2016). Indeed, blocking of QD655Sav with either free biotin or biotinylated IDT785 effectively prevented Sav-mediated B4F quenching, suggesting that the biotin end of IDT785 was readily available for QD655Sav binding (Figure 3).

Molecular mechanisms of SSRI interactions with SERT have been of great interest in the pursuit of drugs with greater specificity and fewer adverse side effects. As a result of extensive efforts, X-ray and cryo-EM structures of SSRI-bound SERT are now publicly available (Coleman and Gouaux, 2018). The general consensus is that high-affinity SSRIs occupy the S1 central-binding site composed of subsites A, B, and C and stabilize the transporter in an outward-facing conformation, although ambiguity remains concerning the precise binding poses of SSRIs within S1 (Coleman et al., 2020). A secondary, allosteric site S2 is formed above S1 in the extracellular vestibule of the transporter in the outward-facing conformation, permitting modulation of S1 ligand dissociation kinetics. Previously, docking of 3-(1,2,3,6-tetrahydropyridin-4-yl)-1H-indole derivative to the vestibular binding pocket of hSERT model based on the bacterial homolog LeuT showed that the indole docked deep into the vestibular pocket and the S2 substrate site, whereas tetrahydropyridine N-substituents extended out to the extracellular environment (Nolan et al., 2011). Surprisingly, molecular docking performed via user-friendly Autodock Vina in UCSF Chimera revealed that the best-scoring poses of the parent drug of IDT785 attached to the phenylethyl spacer and a short PEG_2_ chain were oriented in such manner that the binding site of the indole moiety overlapped with the central binding site occupied by paroxetine in the cryo-EM SERT structure and the tetrahydropyridine N-substituent extended into the extracellular space, thus supporting the importance of tetrahydropyridine indole portion for SERT binding and consequently enabling capture of extracellular biotin. A separate cluster of lower-scoring binding poses was located in the outer extracellular vestibule partially formed by EL2 and EL4 of SERT. Extending distal PEG_2_ spacer to PEG chain with the number of repeat units *n* ≥ 3 resulted in a loss of indole access to the central binding site and the best-scoring binding poses (<7 kcal/mol; data not shown) located in the outer extracellular vestibule. This suggests that the introduction of a longer PEG spacer might prevent the indole moiety from accessing deeper into the extracellular vestibule and substrate sites S1 and S2, thus resulting in modest SERT binding affinity as determined in IDT307-based uptake inhibition experiments.

Since biological activity and availability for binding of both ends of IDT785 was verified, the next step was to assess whether IDT785 facilitated specific QD labeling of surface SERT proteins. To this end, wild-type hSERT and YFP-tagged hSERT constructs were transiently expressed in HEK-293T cells and then labeled with biotinylated IDT785 (10 μM or ∼1.4 × its IC_50_ value) and 0.1 nM QD655Sav stepwise; subnanomolar QD concentration was chosen as non-specific binding of commercially available streptavidin-conjugated QDs is well-controlled below 1 nM (Bentzen et al., 2005; Rosenthal et al., 2011) and is thus preferred for single QD tracking assays. Imaging of hSERT-expressing cells revealed sparse QD labeling associated with the plasma membrane that was considerably blocked in the presence of paroxetine (Figure 5). This observation was confirmed by imaging of HEK-293T cells transiently expressing YFP-tagged hSERT and incubated with IDT785 and QD655Sav at identical conditions (Figure 6). YFP-SERT signal was particularly useful for identifying a fraction of QDs non-specifically adsorbed to the coverslip. Minimal non-specific binding of IDT785-QDs to the coverslip and plasma membrane in paroxetine-blocked cells was observed, likely due to the synergistic effect of the reduced surface area, length and hydrophobicity (logP) of the phenethyl spacer compared to the undecyl linker in our earlier ligands. Interestingly, YFP-SERT was not uniformly distributed in the plasma membrane at the coverslip interface when imaged either via spinning disk confocal microscope (Figure 6) or TIRF microscope (Figure 7). This phenomenon allowed us to generate binary maps of YFP-SERT cell surface distribution and quantitate the extent of QD labeling specificity by determining individual QD localization to the YFP-SERT-rich membrane regions. As a result, the total number and fraction of detected QDs that were colocalized with YFP-SERT were significantly greater for transfected HEK-293T cells in the absence of paroxetine compared to paroxetine-blocked cells. Quantitative confirmation of labeling specificity at these conditions (10 μM IDT785 for 10 min; 0.1 nM QD655Sav) allowed us to proceed toward single QD tracking experiments.

Single-molecule fluorescence microscopy has revealed that lateral diffusion is a fundamental feature of post-translational regulation of various transmembrane receptors, transporters, and ion channels (Rosenthal et al., 2011; Kusumi et al., 2014; Kovtun et al., 2018). Our previous QD-based studies of SERT surface dynamics demonstrated that endogenous SERT was preferentially compartmentalized to cholesterol-rich microdomains and was readily mobilized in response to cholesterol depletion and phosphorylation of its intracellular domain in immortalized serotonergic rat neurons and cultured rat dorsal raphe neurons (Chang et al., 2012b; Bailey et al., 2018). QD tracking in stably transfected CHO cells showed that the ASD-associated, hyperphosphorylated G56A hSERT variant exhibited ∼40% faster diffusion rate at the cell surface (Kovtun et al., 2018). To demonstrate that IDT785 enables QD tracking of YFP-SERT, HEK-293T expressing YFP-tagged transporters were labeled with a combination of IDT785 (500 nM) and QD655Sav (0.05 nM). QD movement was monitored for 600 frames at the rate of 10 Hz, and single QD trajectories were reconstructed from the acquired time-lapse image series (Figure 8). A wide range of diffusive behavior of YFP-SERT was readily evident from the shape of reconstructed QD trajectories, and the RD analysis of MSD-Δt curves in comparison to simulated Brownian motion trajectories showed that 85% of QD trajectories were mobile versus 15% of immobilized QDs, although it was not possible to distinguish QDs non-specifically adsorbed to the coverslip and QDs bound to immobile YFP-SERT (Dahan et al., 2003). The slight majority of mobile QD trajectories exhibited simple Brownian diffusion (45%; median D = 0.04 μm^2^/s), whereas 40% of mobile tracks were determined to undergo restricted diffusion (40%; median D = 0.02 μm^2^/s), consistent with our previous observation that a large pool of endogenous SERT in the neuronal membrane is confined to cholesterol-rich membrane microdomains (Chang et al., 2012b; Bailey et al., 2018). Naturally, an important question arises - how accurately do QDs measure the rate of lateral diffusion of membrane SERT proteins? In fact, suitability of commercially available QDs for tracking surface dynamics of neuronal receptors and transporters has recently been under scrutiny, as improvements in organic fluorophore photophysics, instrumentation, and stochastic activation-based imaging modalities have simplified implementation of dye-based tracking (Lavis, 2017; Shen et al., 2017). For instance, Lee et al. (2017) discovered that over 90% of AMPA glutamate receptors bound to commercially available QDs (diameter > 20 nm) were extrasynaptic and highly mobile, whereas ∼90% of AMPA receptors labeled with smaller dyes and compact QDs were diffusing in a confined manner in nanodomains within the post-synaptic density (PSD). As another example, Abraham et al. (2017) reported that although QDs could resolve large differences in B cell receptor mobility, larger QDs compared to a small cyanine dye (Cy3) sterically hindered receptor mobility at the cell-coverslip interface, altered the frequency of transitions between fast and slow diffusion states, and reduced the size of transient confinement zones. Recently, ensemble fluorescence recovery after photobleaching (FRAP) measurements performed on monomeric GFP fused to the amino terminus of hSERT heterologously expressed in HEK-293 cells yielded a mobile fraction of 82 ± 8%, with a population diffusion coefficient of 0.151 ± 0.003 μm^2^/s estimated via direct tracking of individual mGFP-SERT (Anderluh et al., 2017). Single-molecule tracking of mGFP-SERT in transiently transfected CHO cells revealed a major mobile fraction of transporters (85%) characterized by D_1_ = 0.30 μm^2^/s and a minor pool (15%) of mGFP-SERT immobilized during the observation period with D_2_ = 0.05 μm^2^/s (Anderluh et al., 2014). To perform direct comparison, we recorded lateral movement of individual dim YFP-SERT spots in the TIRF mode in HEK-293T cells 6 h post-transfection to ensure low expression density suitable for single molecule tracking. The majority of tracks (63% of 7802 tracks) were determined to be mobile as defined by the previously used D_cutoff_ = 5 × 10^–4^ μm^2^/s with a lateral diffusion rate (median D) of 0.07 μm^2^/s (Δt of 50 ms; Supplementary Figure 5). The diffusion rate of the mobile YFP-SERT pool 6 h post-transfection was comparable to that of the mobile QD-tagged YFP-SERT pool imaged ≥ 24 h post-transfection in our study (D_median, YFP_ = 0.066 μm^2^/s [0.017–0.16 IQR]; D_median, Qdot_ = 0.030 μm^2^/s [0.015–0.051 IQR]; ^∗∗∗^*p* < 0.001, Mann-Whitney *U* test). It should be noted that the diffusion coefficient values in our studies were nearly an order of magnitude lower that previously reported average diffusion rates for mGFP-SERT (Anderluh et al., 2014, 2017). Several factors might contribute to the variability in the diffusion rate measurement. Higher photon output per frame at lower acquisition frame rates improves the localization accuracy of slowly moving molecules, resulting in significantly smaller reported diffusion coefficients for slower molecules (Lee et al., 2019). It is also possible that antagonist-conjugated QDs target a membrane pool of YFP-SERT that is “transport-willing” and distinct from tracked YFP-SERT or mGFP-SERT species. In addition to the probe potentially altering the transporter conformational equilibrium, a ligand-based approach may also lead to changes in constitutive transport trafficking to and from the plasma membrane. In fact, SERT was reported to undergo constitutive endocytosis in transfected CAD and HEK-293 cells, with a modest surface loss under basal conditions (up to 10% over 1 h at 37°C) when assessed via internalization assays based on the antibody feeding, surface ELISA, and fluorescent cocaine analog labeling (Rahbek-Clemmensen et al., 2014). However, Kittler et al. demonstrated that antagonists and substrates differentially regulated SERT endocytosis in serotonergic neurons over the course of 4 h (Kittler et al., 2010). In particular, **fluoxetine** led to a dose-dependent internalization of SERT (1 μM: to 63.8 ± 2.5% of control; 10 μM: to 33.9 ± 1.9% of control); **paroxetine** induced a dose-independent downregulation of SERT (500 nM: 75.5 ± 5.9; 10 μM: 66.0 ± 6.7% of control); **sertraline** induced SERT internalization (1 μM: 75.8 ± 2.2%; 5 μM: 61.9 ± 1.8%), while exposure to 50 μM **cocaine** significantly enhanced SERT surface expression (123 ± 3.9%) compared to control levels. Application of substrates led to SERT internalization as follows: **5-HT**, 1 μM: 64.7 ± 2.9% and 10 μM: 57.7 ± 1.9%; **MDMA**, 43.4 ± 1.6%. Additionally, Jørgensen et al. (2014) demonstrated 5-HT-induced reduction in SERT surface expression in both stably transfected HEK-293 cells and cultured raphe serotonergic neurons. When we assessed SERT-bound Qdot localization with respect to the plasma membrane labeled with Cell Mask Deep Red (Supplementary Figure 6) during the typical time course of a labeling/tracking experiment, we observed minimal intracellular Qdot accumulation. Thus, minimizing time elapsed between the final wash steps post-labeling and time-lapse image series acquisition likely allows us to capture the surface trafficking events.

Another factor that confounds interpretation of method-to-method differences in SERT diffusion rate is the irregular pattern of YFP-SERT distribution in the plasma membrane of HEK-293T cells observed in our spinning disk confocal and TIRF images. Analysis of SIM images of YFP-SERT distribution corroborated our preliminary observation that YFP-SERT density was markedly increased in the membrane edges and protrusions (Figure 9). This phenomenon is similar to conformation-dependent preferential accumulation of dopamine transporter in membrane protrusions (filopodia) that was previously observed in several neuronal and non-neuronal heterologous expression hosts (Caltagarone et al., 2015; Kovtun et al., 2019). QD tracking of YFP-SERT localized to membrane protrusions revealed a simple Brownian, unrestricted pattern of diffusion that resembles surface dynamics of the extrasynaptic pool of neuronal receptors (Maynard and Triller, 2019). Physiological significance of SERT preferential targeting to protrusions and its unhindered lateral mobility within these regions is unclear. Ultrastructural studies demonstrated that SERT primarily resided in the extrasynaptic compartments along the axonal projections in the vicinity of asymmetric synapses (Zhou et al., 1998; Tao-Cheng and Zhou, 1999; Miner et al., 2000); thus, to draw parallels with our state-of-the-art knowledge of neuronal receptor surface dynamics, lateral mobility might represent a yet unappreciated dynamic mechanism of SERT delivery and stabilization in the vicinity of synaptic junctions *in vivo*, where SERT is optimally positioned to clear synaptically released serotonin.

In conclusion, this work presents synthesis, extensive characterization, and QD-based imaging application of a novel biotinylated, PEGylated 3-(1-phenylethyl-1,2,3,6-tetrahydropyridin-4-yl)-1H-indole (IDT785) ligand. Biological activity of IDT785 was demonstrated by its ability to inhibit hSERT-mediated IDT307 uptake with IC_50_ = 7.2 ± 0.3 μM and through the efficient capture of its biotin terminus by streptavidin-conjugated QDs in solution. As a result, IDT785 enabled specific QD labeling of surface WT and YFP-tagged SERT proteins heterologously expressed in HEK-293T cells. IDT785-based single QD tracking of YFP-SERT membrane dynamics allowed classification of the membrane pool of transporters into distinct fractions undergoing simple Brownian, restricted, or immobilized diffusion. Moreover, IDT785-enabled QD tracking resulted in characterization of membrane diffusion of YFP-SERT proteins preferentially localized to the membrane edges and protrusions in HEK-293T cells. Our next goals are (1) to determine whether IDT785 structure can be optimized to achieve sub-μM SERT binding affinity and (2) to evaluate if IDT785 enables specific QD labeling and tracking of endogenous SERT proteins. Overall, our work provides a generalizable blueprint for developing and rigorous characterization of biotinylated ligands that enable QD-based detection of transmembrane neuronal proteins. Our ultimate goal is to implement this approach in the native neuronal context, and we anticipate further synthetic optimizations toward the development of a high-affinity, specific, compact Qdot probe that would allow us to detect SERT and to monitor its surface dynamics *in situ*. We envision that tracking SERT in intact tissue would shed light on intrinsic differences in spatially segregated compartments of raphe 5-HT neurons, which display highly branched axonal processes with numerous axonal-like varicosities. We are particularly interested in SERT diffusion with respect to the axon initial segment that may serve as a physical barrier to separate somatodendritic and axonal SERT pools. We also aim to answer the question whether stimulation is capable of inducing transient SERT stabilization in the vicinity of presynaptic boutons, ultimately leading to altered 5-HT clearance rates.

## Data Availability Statement

The raw data supporting the conclusions of this article will be made available by the authors, without undue reservation.

## Author Contributions

IT, OK, RT, LB, and TJ performed the experiments and analyzed data. IT, OK, RT, LB, and SR conceived the study, designed the experiments, and wrote the manuscript. All authors contributed to the article and approved the submitted version.

## Conflict of Interest

The authors declare that the research was conducted in the absence of any commercial or financial relationships that could be construed as a potential conflict of interest.

## Publisher’s Note

All claims expressed in this article are solely those of the authors and do not necessarily represent those of their affiliated organizations, or those of the publisher, the editors and the reviewers. Any product that may be evaluated in this article, or claim that may be made by its manufacturer, is not guaranteed or endorsed by the publisher.

## Footnotes

1 http://n2t.net/addgene:70103; RRID:Addgene_70103
