# Supplementary material for: A Novel Biotinylated Homotryptamine Derivative for Quantum Dot Imaging of Serotonin Transporter in Live Cells
Source: Front Cell Neurosci. 2021 Nov 18;15:667044. doi: 10.3389/fncel.2021.667044 (PMC8637195; doi:10.3389/fncel.2021.667044)
Supplement: Supplementary file 1 [file Data_Sheet_1.DOCX]

Supplementary Material


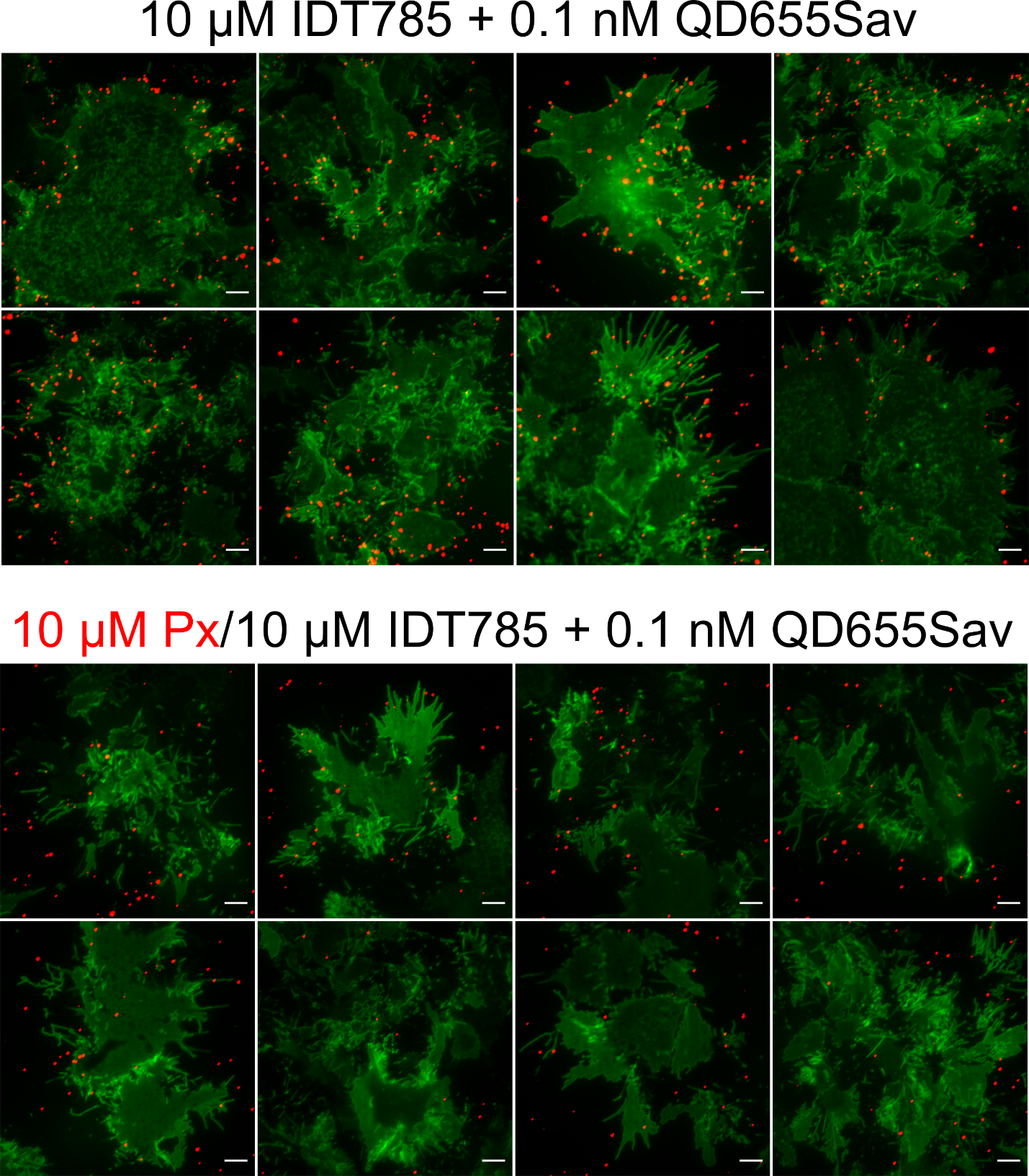


**Supplementary Figure 1**. QD TIRF imaging of YFP-SERT in transfected HEK-293T cells at the membrane-coverslip interface. Top and bottom panels show representative images of YFP-SERT-HEK-293T cells sequentially labeled with 10 μM IDT785 and 0.1 nM QD655Sav in the absence or presence of 10 μM paroxetine, respectively. Scale bar: 5 μm.


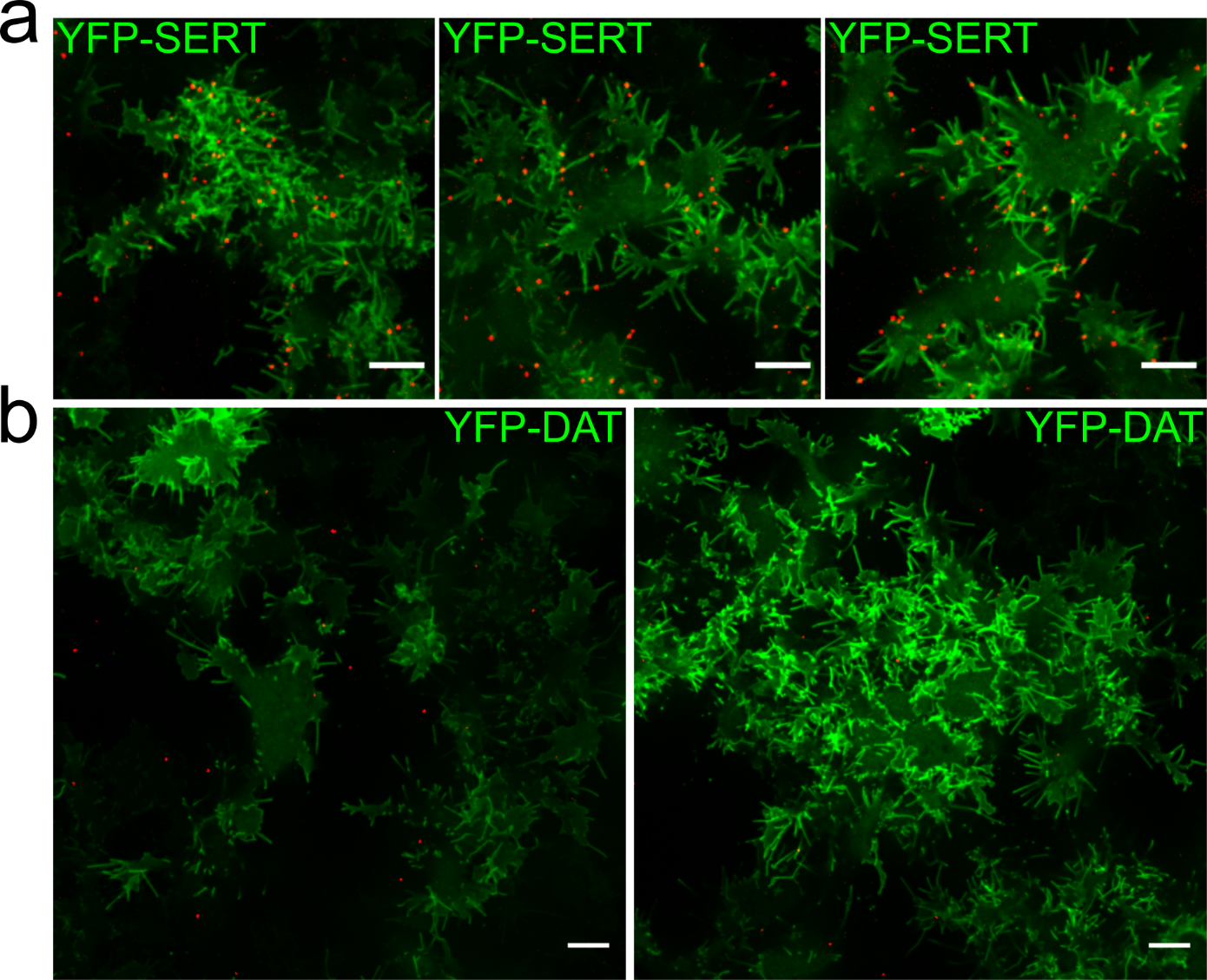


**Supplementary Figure 2**. Spinning disk confocal imaging of QD labeling of YFP-SERT and YFP-DAT (control) in transfected HEK-293T cells. (a) Representative images of YFP-SERT-HEK-293T cells sequentially labeled with 500 μM IDT785 and 0.05 nM QD655Sav are shown. The obtained QD labeling density is suitable for single quantum dot tracking. Scale bar: 10 μm. (b) Representative larger field of view images of YFP-DAT-HEK-293T cells sequentially labeled with 500 μM IDT785 and 0.05 nM QD655Sav are shown. Lack of cell-associated QD labeling confirms IDT785 specific binding to SERT. Scale bar: 10 μm.


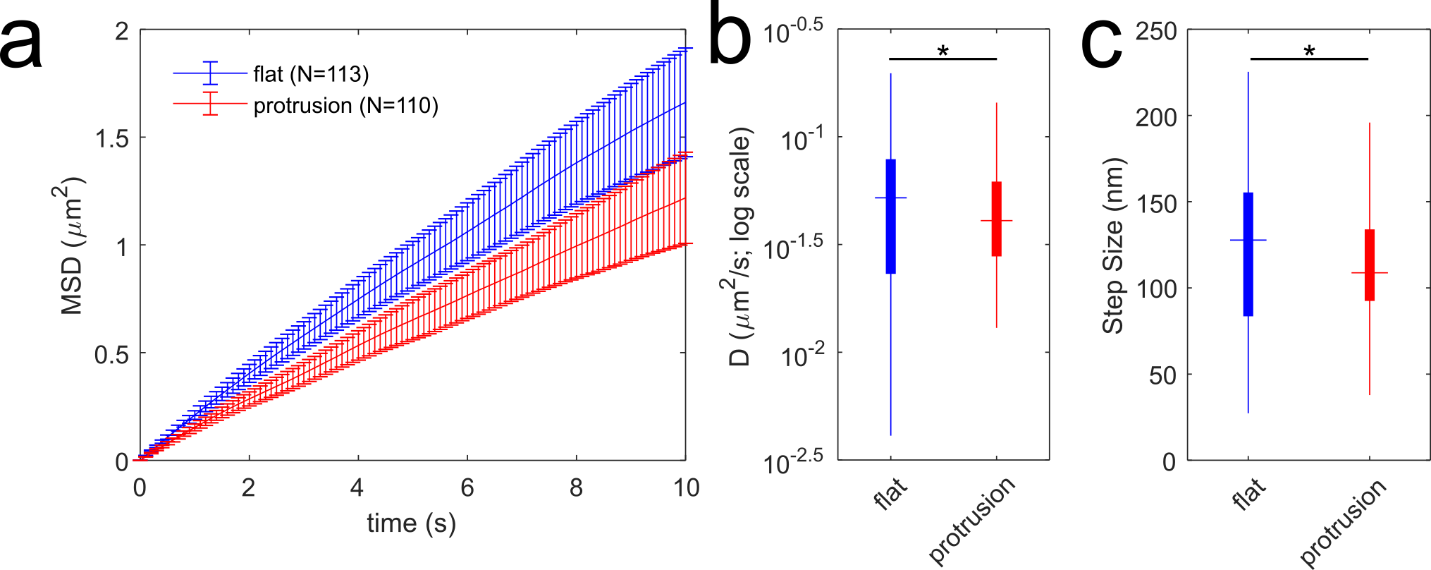


**Supplementary Figure 3**. Diffusion analysis of QD-labeled YFP-SERT in distinct membrane regions of HEK-293T cells . a) Time-dependent averaged MSD curves are shown for YFP-SERT-QD trajectories that are localized to flat membrane zones (113 tracks from 3 independent experiments) and membrane protrusions (110 tracks from 3 independent experiments). MSD curves include weighted standard deviation error bars. (b) Box plot shows the diffusion coefficient distribution for each pool of trajectories. The median value is shown as the colored horizontal line in the box, the 25-75% IQR interval corresponds to the length of the colored box, and whiskers extend to 5% and 95% percentile values. The distributions were compared using the Mann-Whitney U test (*p<0.05, flat membrane: D_median_ = 0.052 μm^2^/s, IQR = [0.024, 0.078]; protrusions: D_median_ = 0.041 μm^2^/s, IQR = [0.028, 0.061]). (c) Box plot shows the instantaneous step size (per Δt = 100 ms) distribution for trajectories of each membrane region. The median value is shown as the colored horizontal line in the box, the 25-75% IQR interval corresponds to the length of the colored box, and whiskers extend to 5% and 95% percentile values. The distributions were compared using the Mann-Whitney U test (*p<0.05, flat membrane: step size_median_ = 128 nm, IQR = [84, 155]; protrusions: step size_median_ = 109 nm, IQR = [92, 134]).


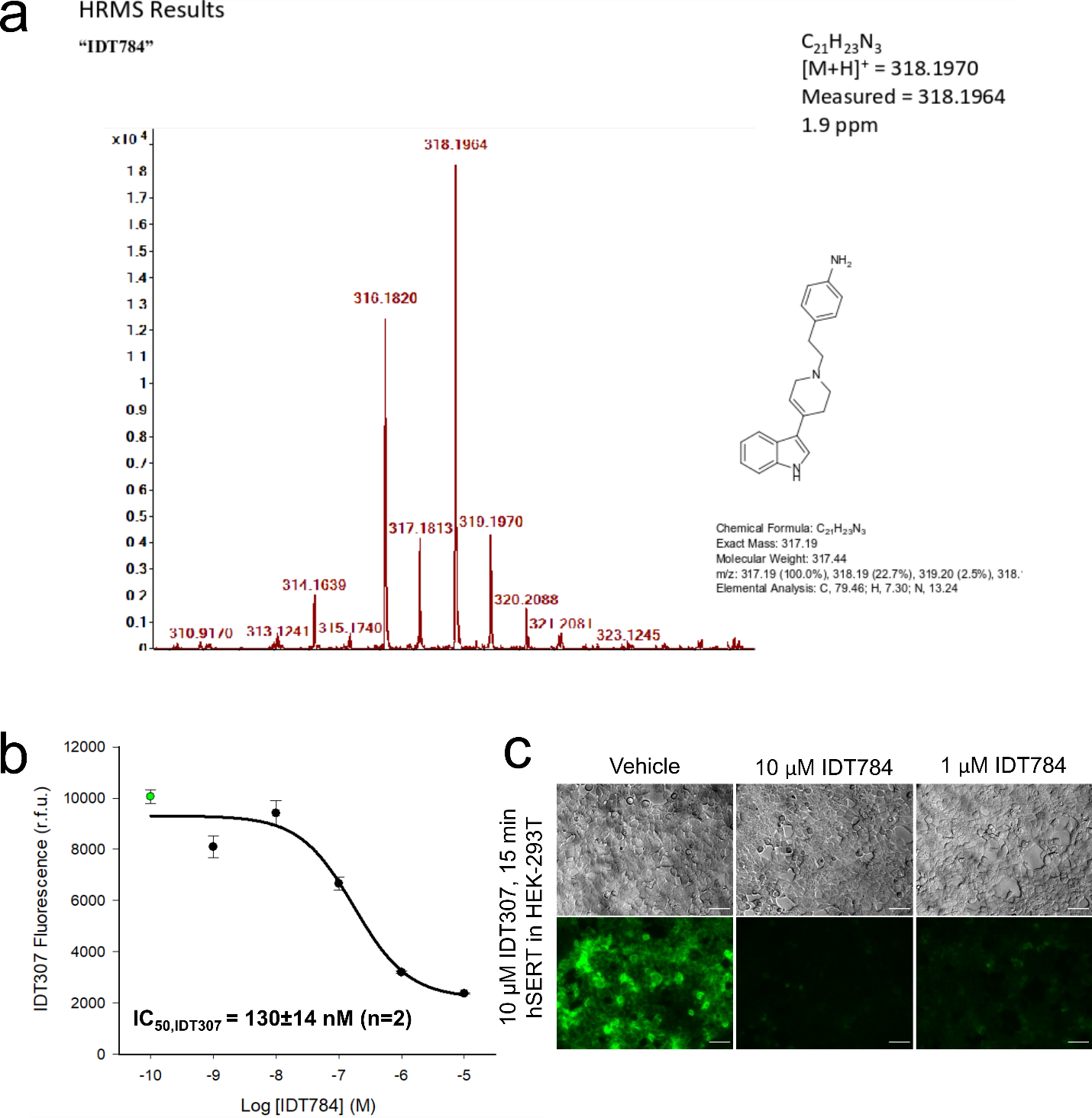


**Supplementary Figure 4**. Characterization of the drug intermediate IDT784 prior to PEGylation. (a) A high-resolution mass spectrometry confirmation of the IDT784 structure. (b) IDT307 transport assay was used to determine the IC_50_ value for IDT784. (c) Fluorescence images were acquired in parallel to complement the microplate reader data used to construct the inhibition curves in b. Scale bar: 20 μm.


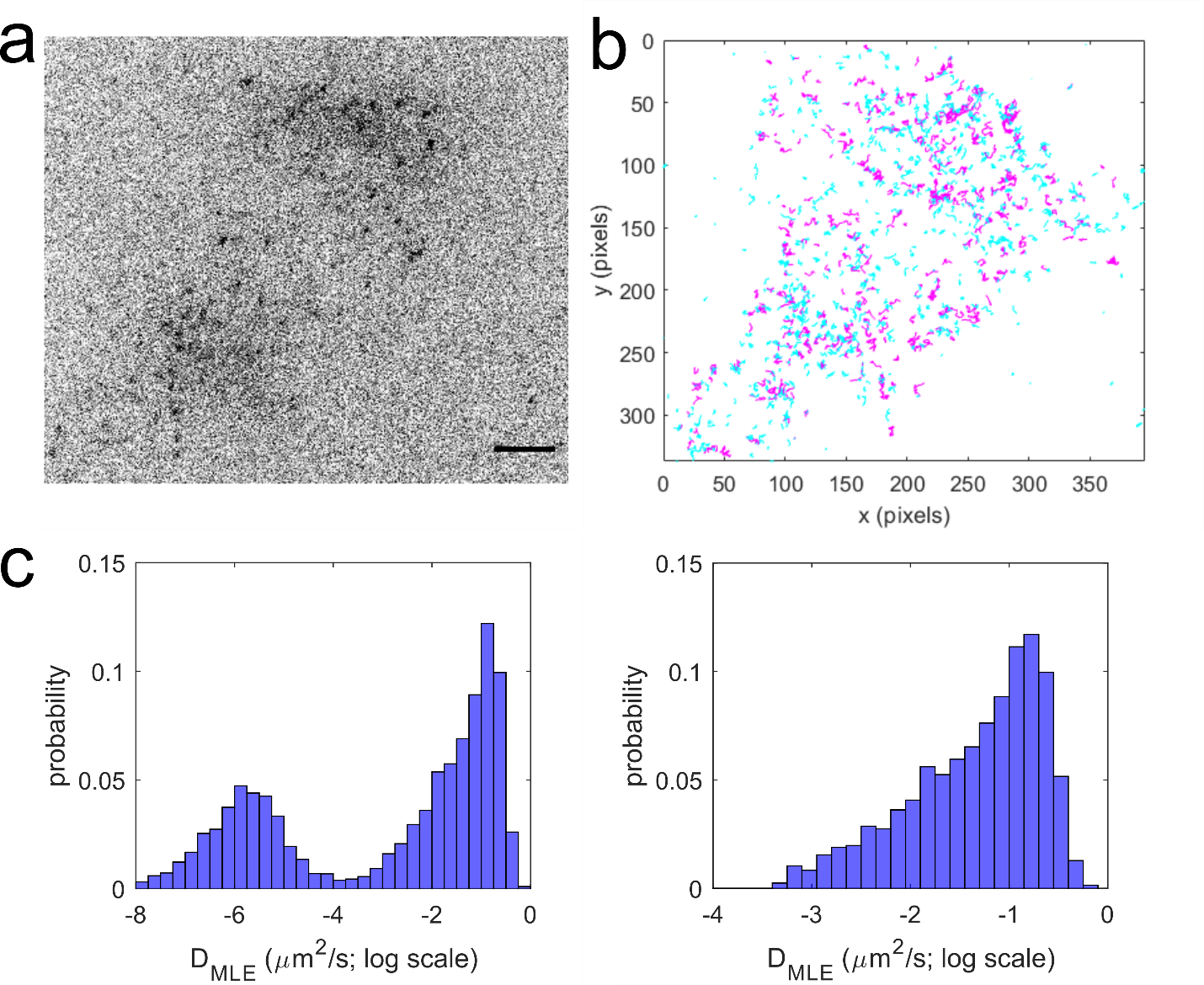


**Supplementary Figure 5**. Single particle tracking of individual YFP-SERT puncta in live HEK-293T cells. (a) A representative image demonstrates that individual YFP-SERT puncta could be detected at the membrane-coverslip interface in the TIRF mode. Scale bar: 5 μm. (b) YFP-SERT trajectories were reconstructed in the TrackMate ImageJ plugin and plotted (minimum length = 5 steps, Δt = 50 ms, maximum gap between frames = 2, maximum displacement between frames = 5 pixels, 1 pixel = 108 nm). (c) A histogram of the diffusion coefficient (D­_MLE_) distribution is shown (total tracks = 7802 from two independent imaging sessions). The median D_MLE_ of the mobile pool (threshold D_MLE_ > 5 × 10^-4^ μm^2^/s) was found to be 0.066 μm^2^/s (25-75% IQR [0.017, 0.16]).

**
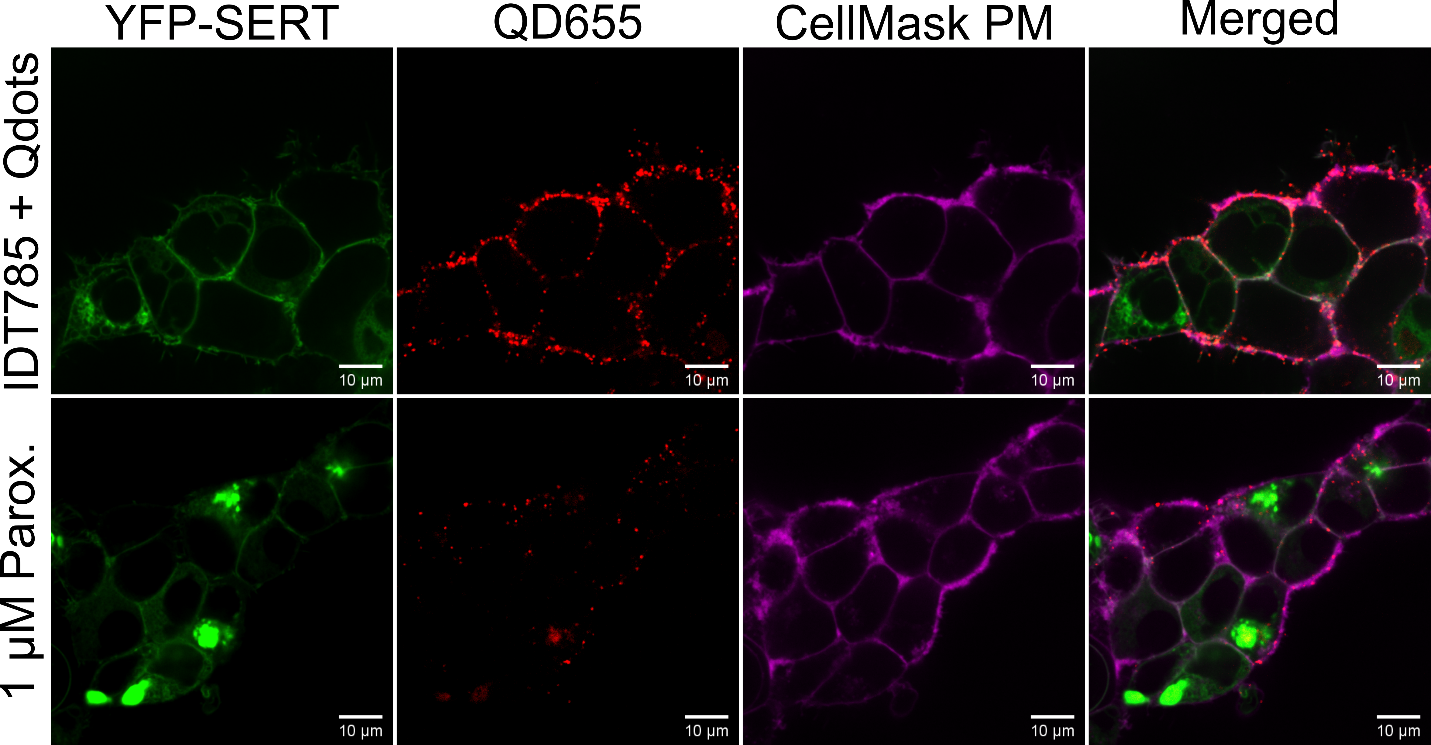
**

**Supplementary Figure 6**. Verification of surface YFP-SERT Qdot detection with IDT785. Briefly, HEK-293T cells expressing YFP-SERT construct 24 hours after transfection were labeled with 10 μM IDT785 for 20 min, 0.5 nM QD655Sav in DMEM Fluorobrite/1% BSA for 5 min with CellMask™ Plasma Membrane Deep Red spiked into the QD labeling solution at 1:20,000 dilution. Control cells were preblocked with 1 μM paroxetine for 20 minutes. YFP molecules were excited using the 488-nm laser and the emission was collected via the 525 nm (+/- 18 nm) emission filter. QD655 were excited using the 405-nm laser and the emission was collected via the 641 nm (+/- 75 nm) emission filter. Cell Mask™ PM Deep Red was excited using the 647-nm laser and the emission was collected via the 700 nm (+/- 37 nm) emission filter. Qdot localization to the membrane outlined with the Cell Mask and the absence of intracellular Qdot accumulation were apparent.
